# Exploring sample preparation and data evaluation strategies for enhanced identification of host cell proteins in drug products of therapeutic antibodies and Fc-fusion proteins

**DOI:** 10.1007/s00216-020-02796-1

**Published:** 2020-07-20

**Authors:** Wolfgang Esser-Skala, Marius Segl, Therese Wohlschlager, Veronika Reisinger, Johann Holzmann, Christian G. Huber

**Affiliations:** 1 Bioanalytical Research Labs, Department of Biosciences, University of Salzburg, Hellbrunner Straße 34, 5020 Salzburg, Austria; 2 Christian Doppler Laboratory for Innovative Tools for Biosimilar Characterization, University of Salzburg, Hellbrunner Straße 34, 5020 Salzburg, Austria; 3 Technical Development Biosimilars, Global Drug Development, Novartis, Sandoz GmbH, Biochemiestraße 10, 6250 Kundl, Austria

**Keywords:** Host cell proteins, Monoclonal antibodies, Tandem mass spectrometry, Probabilistic protein inference, Biopharmaceutical

## Abstract

Manufacturing of biopharmaceuticals involves recombinant protein expression in host cells followed by extensive purification of the target protein. Yet, host cell proteins (HCPs) may persist in the final drug product, potentially reducing its quality with respect to safety and efficacy. Consequently, residual HCPs are closely monitored during downstream processing by techniques such as enzyme-linked immunosorbent assay (ELISA) or high-performance liquid chromatography combined with tandem mass spectrometry (HPLC-MS/MS). The latter is especially attractive as it provides information with respect to protein identities. Although the applied HPLC-MS/MS methodologies are frequently optimized with respect to HCP identification, acquired data is typically analyzed using standard settings. Here, we describe an improved strategy for evaluating HPLC-MS/MS data of HCP-derived peptides, involving probabilistic protein inference and peptide detection in the absence of fragment ion spectra. This data analysis workflow was applied to data obtained for drug products of various biotherapeutics upon protein A affinity depletion. The presented data evaluation strategy enabled in-depth comparative analysis of the HCP repertoires identified in drug products of the monoclonal antibodies rituximab and bevacizumab, as well as the fusion protein etanercept. In contrast to commonly applied ELISA strategies, the here presented workflow is process-independent and may be implemented into existing HPLC-MS/MS setups for drug product characterization and process development.

**Electronic supplementary material:**

The online version of this article (10.1007/s00216-020-02796-1) contains supplementary material, which is available to authorized users.

**Figure:**
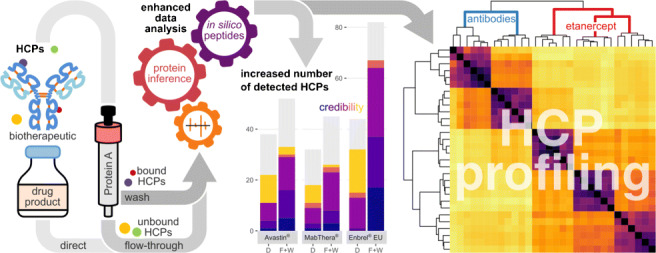
Graphical abstract

## Introduction

Therapeutic monoclonal antibodies (mAbs) and Fc-fusion proteins are conventionally produced in mammalian expression systems such as Chinese hamster ovary (CHO) or human embryonic kidney cells. Naturally, these cells express not only the recombinant target protein but also a plethora of endogenous proteins essential for cellular growth and viability. Despite rigorous clean-up procedures during downstream processing, minor amounts of these host cell proteins (HCPs) may be co-purified with the therapeutic protein and remain in the final drug product (DP) [1–5]. Since these contaminating proteins may affect DP quality [6–12] or provoke immune responses when the drug is administered [13, 14], HCPs are generally considered in the context of critical quality attributes (CQAs) and product quality attributes (PQAs) [15]. Hence, sensitive and reliable analytical procedures for identifying and quantifying these impurities are indispensable for the production and release of biopharmaceuticals. Although there are no general guidelines specifying maximum acceptable HCP loads in DPs, manufacturers commonly aim at amounts below 1 to 100 ng of HCP per mg of drug substance (DS) in the final product (i.e., 1 to 100 ppm) [16]. Consequently, challenges in HCP characterization arise from low amounts of the contaminating proteins at large excess of DS, thus requiring a wide dynamic range to be covered by analytical methods.

To date, enzyme-linked immunosorbent assay (ELISA) represents the gold standard for HCP detection and quantification. In this context, polyclonal antibodies raised against the supernatant of null cells, i.e., cells that do not express the product gene, are used as primary antibodies for HCP detection [17, 18]. Due to its robustness and simplicity, ELISA can be performed in a high-throughput manner [19]. In addition, common cell lines and similar upstream conditions allow the development of platform assays that can be used for analyzing multiple DPs [20–22]. Yet, sensitivity of ELISA depends on the immunogenicity of each individual protein in the null cell supernatant, thus hindering relative quantification of different HCPs [23].

As an alternative, HCP analysis based on high-performance liquid chromatography (HPLC) combined with tandem mass spectrometry (MS/MS) has emerged [24, 25]. HPLC-MS/MS-based methods are orthogonal to ELISA in that they are independent from specific antibodies for detection. These workflows involve proteolytic digestion of HCP-containing samples and subsequent HPLC-MS/MS analysis. Fragment ion spectra of HCP peptides allow protein identification based on comparison with theoretical spectra derived from an HCP sequence database. Thus, HPLC-MS/MS-based workflows outperform ELISA in that they provide information on individual HCP identities and amounts rather than a total HCP content [26]. However, their main drawback lies in a dynamic range limited to three to four orders of magnitude, while analysis of low-abundant HCPs requires up to six orders of magnitude considering the DS to HCP ratio [27]. In addition, co-elution of DS- and HCP-derived peptides may result in ion suppression of low-abundant HCP peptides, preventing detection of the corresponding contaminant [28].

To overcome these limitations, two strategies have been described. On the one hand, multidimensional HPLC setups have been implemented to tackle sample complexity, thereby offering lower limits of detection and quantification [29–31]. However, these setups suffer from low throughput as well as limited robustness and reproducibility, which hampers their application in quality control [32, 33]. On the other hand, protein A affinity chromatography may be exploited to deplete Fc domain-containing DS, i.e., mAbs and Fc-fusion proteins, by highly specific interaction between protein A and the Fc domain before tryptic digestion and HPLC-MS/MS analysis [34, 35]. This enables identification of HCPs with abundances in the low ppm range applying a single chromatographic dimension [36, 37]. Moreover, wash solutions containing various additives may be applied to the DS captured on the protein A column to facilitate elution and subsequent analysis of HCPs that interact with the DS or the affinity resin [38–40]. Alternatively, enrichment of HCPs by applying a molecular weight cutoff filtration step has recently been described [41].

Previous studies tended to describe optimization of laboratory protocols with respect to the number of HCPs identified, while acquired MS/MS data was frequently analyzed using standard settings. Here, we describe an optimized data evaluation protocol that enhances process-independent HCP identification based on established analytical techniques, i.e., DS depletion via protein A affinity chromatography followed by reversed phase-HPLC-MS/MS. This data evaluation protocol combines (a) probabilistic protein inference based on all peptides identified from fragment ion mass spectra with (b) peptide detection on the full-scan MS level, i.e., even in the absence of MS/MS spectra. We demonstrate generic applicability of our workflow across Fc domain-containing biotherapeutics by assembling HCP profiles for a panel of structurally diverse, commercial grade DPs. Furthermore, a comparative analysis of non-depleted DPs reveals synergistic benefits of our data evaluation protocol and the depletion workflow for HCP identification. Thus, our approach represents a powerful tool that may be implemented into existing HPLC-MS/MS setups for DP characterization as well as in the context of process development.

## Materials and methods

### Materials

Acetonitrile (≥ 99.9%) was purchased from VWR International (Vienna, Austria). Tris(2-carboxyethyl)phosphine hydrochloride (TCEP, ≥ 98.0%), iodoacetamide (≥ 99.9%), formic acid (98.0–100%), guanidine hydrochloride (≥ 99%), disodium phosphate dihydrate (HNa_2_PO_4_·2H_2_O, ≥ 99.0%), tetramethylammonium chloride (TMAC, ≥ 99.0%), glycine (≥ 99.0%), l-arginine monohydrochloride (≥ 98%), sodium chloride (NaCl, ≥ 99.5%), ammonium bicarbonate (LC-MS grade), ethanol (LC-MS grade), *E. coli β*-galactosidase, and bovine *β*-lactoglobulin were obtained from Sigma-Aldrich (Vienna, Austria). Tris(hydroxymethyl)aminomethane (Tris, ≥ 99%) was purchased from SERVA Electrophoresis (Heidelberg, Germany). Trypsin (Mass Spec Grade, V5111) was obtained from Promega (Madison, WI, USA). The Hi3 *E. coli* standard, consisting of six synthetically prepared, highly ionizing peptides derived from *E. coli* chaperone protein ClpB, was purchased from Waters (Milford, MA, USA). For all experiments, ultrapure water produced in-house by a Millipore Integral 3 unit (Merck/Millipore, Billerica, MA, USA) was used.

The following DPs were examined: MabThera® (batch H0139B01 expiring 01/2016, 10 mg mL^−1^ rituximab) and Avastin® (batch B7214HO9 expiring 03/2018, 25 mg mL^−1^ bevacizumab) from F. Hoffmann-La Roche Ltd. (Basel, Switzerland); Enbrel® (batches E11132 expiring 03/2011 and 1040542 expiring 03/2016, 50 mg mL^−1^ etanercept) from Pfizer (New York, NY, USA); and Benepali® (batch CT0056 expiring 09/2018, 50 mg mL^−1^ etanercept) from Samsung Bioepis UK Limited.

### Drug substance depletion by protein A affinity chromatography

**Fig. 1:**
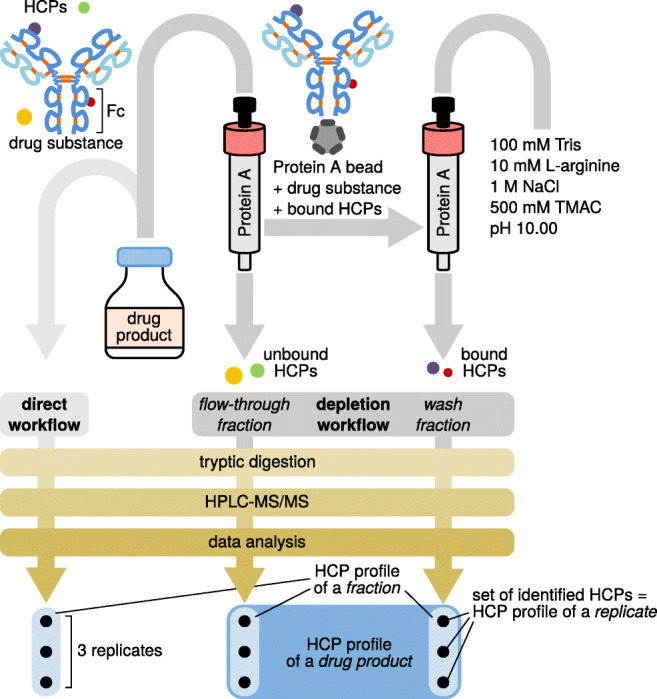
Schematic representation of the workflows used for HCP discovery in a drug product, which comprises a drug substance (i.e., the therapeutic protein) and minute amounts of HCPs. The two strategies applied involve direct analysis of HCPs in drug products or analysis of fractions obtained upon affinity depletion of the Fc domain-containing therapeutic protein. Peptide mixtures obtained upon tryptic digestion were analyzed by HPLC-MS/MS in triplicate. Data evaluation yields HCP profiles of replicates, which may be aggregated to HCP profiles of fractions and, ultimately, drug products, as indicated at the bottom of the figure

DPs were equilibrated to room temperature (25 °C). Twenty milligrams of each DP, 2 μg *β*-galactosidase, and 2 μg *β*-lactoglobulin were brought to a final volume of 2.0 mL using 20 mM Na_2_HPO_4_, pH 7.30, resulting in a final concentration of 10 mg mL^−1^. Affinity chromatography was performed at 25 °C using a 5.0-mL Hamilton syringe (1000 series Gastight, PTFE luer lock, Sigma-Aldrich, Vienna, Austria) for column equilibration, sample loading, column washing, and regeneration. For each therapeutic protein, a protein A column (HiTrap MabSelect SuRe, 1 mL, Sigma-Aldrich) was manually equilibrated with 10 mL 20 mM Na_2_HPO_4_, pH 7.30. Samples were loaded onto the column at a flow rate of 100 μL min^−1^ generated by a syringe pump (230 VAC EW-74900-05, Cole-Parmer, Vernon Hills, IL, USA), and the flow-through was collected**.** After rinsing the syringe with 10 mL ultrapure water, 2.0 mL of a wash solution consisting of 10 mM Tris, 100 mM l-arginine, 1.0 M NaCl, and 500 mM TMAC, adjusted to pH 10.00 using HCl, was applied at a flow rate of 100 μL min^−1^. The wash fraction was collected and protein A columns were re-equilibrated with 10 mL 20 mM Na_2_HPO_4_, pH 7.30. The column was then regenerated by elution of bound DS using 5.0 mL 100 mM glycine buffer, pH 2.80. Protein A columns were then flushed with 10 mL 20% (v/v) ethanol and stored at 4 °C. A dedicated protein A column was used for each DP. Figure 1 summarizes the steps of the depletion workflow.

### Sample preparation for peptide analysis

Flow-through and wash fractions collected during protein A affinity chromatography (2.0 mL each) were concentrated to 50 μL using Amicon Ultra 0.5 mL 10 kDa MWCO centrifugal filters (Sigma-Aldrich) at 14000×*g*, 25 °C. Samples were denatured and reduced by addition of 450 μL 6.0 M guanidine hydrochloride and 5.0 μL 500 mM TCEP, respectively, for 1.0 h at 37 °C while shaking at 1000 rpm. After alkylation with 15 μL 500 mM iodoacetamide for 30 min at 25 °C in the dark, the buffer was exchanged to 50 mM ammonium bicarbonate, pH 7.80, using Amicon filters. Five micrograms of trypsin was added to each sample, followed by incubation for 16 h at 37 °C, 1000 rpm. Samples were then fully dried at 45 °C for 2 h at 1000 rpm using a vacuum centrifuge, and subsequently dissolved in 10 μL 0.10% (v/v) aqueous formic acid. For direct HCP identification from DPs, 200 μg of each therapeutic protein, 20 ng *β*-galactosidase, and 20 ng *β*-lactoglobulin were digested with trypsin and dried as described above. Samples were adjusted to a concentration of 4.0 μg μL^−1^ with 0.10% (v/v) formic acid and stored at − 20 °C until analysis. To each sample of the depletion workflow, 250 fmol Hi3 standard were added immediately before injection.

### Peptide analysis by HPLC-MS/MS

HPLC-MS/MS measurements were carried out on a Thermo Scientific™ UltiMate™ 3000 HPLC system with flow splitting (1:100) coupled to a Thermo Scientific™ Q Exactive™ Plus hybrid quadrupole-Orbitrap mass spectrometer (Thermo Fisher Scientific). Both instruments were operated with Thermo Scientific™ Chromeleon™ Chromatography Data System (CDS) version 7.2.6. Tryptic peptides were separated on a 150 × 0.30 mm Waters ACQUITY UPLC® M-Class CSH™ C18 column with 1.7 μm particles (130 Å pore size) at a flow rate of 2.0 μL min^−1^ and constant column temperature of 50 °C. A linear gradient from 2.0 to 35% mobile phase B over 80 min was employed (mobile phase A: 0.10% (v/v) formic acid in ultrapure water; mobile phase B: 0.10% (v/v) formic acid in acetonitrile). At the end of each run, the column was flushed with 100% mobile phase B for 10 min followed by equilibration with 2.0% B for 20 min. The mass spectrometer was operated in positive electrospray ionization mode employing a Thermo Scientific™ Nanospray Flex™ ion source with silica emitter tips (New Objective, Woburn, MA, USA) of 30 μm inner diameter at the orifice. The applied spray voltage corresponded to + 2.50 kV. The transfer capillary was heated to 320 °C and the S-Lens RF level was adjusted to 55. Detection was performed in data-dependent mode with the following settings: mass range 400–2000 *m*/*z*; top 15 method for peptide fragment fingerprinting; one microscan per MS/MS event; dynamic exclusion for precursor selection for 20 s; resolution setting for full MS and MS/MS scans set to 70,000 and 17,500, respectively (defined at 200 *m*/*z*). Peptide fragmentation was induced by higher-energy collision-induced dissociation with a normalized collision energy of 28. Maximum injection time was set to 50 ms for full-scan MS and to 120 ms for MS/MS scans. Automatic gain control target was adjusted to 3 × 10^6^ for full-scan MS and to 2 × 10^4^ for MS/MS scans, respectively, with an underfill ratio of 1%. The isolation width for precursor selection was ± 0.75 *m*/*z*; only precursors with charges between 2+ and 4+ were included.

### Data analysis

HCP identification was accomplished using the Protein Metrics Inc. Byos® v3.4-72-g5fd2d85e63 x64 software, which employs Byonic® v3.4.0 for peptide identification and protein inference, as well as Byologic® v3.4-72-g5fd2d85e63 x64 for quantification. Fragment spectra were searched against a CHO protein sequence database obtained from UniProt (reference proteome UP000001057, last modified on 2019-01-24, downloaded on 2019-07-01, containing 23,885 sequences), to which the following sequences were added: porcine trypsin (UniProt ID P00761), *S. aureus* protein A (P38507), bovine *β*-lactoglobulin (P02754), *E. coli β*-galactosidase (P00722), the Hi3 standard peptide sequences, and the respective DP sequence; thus, there was a distinct database for each DP, and each database contained 23,891 entries. Search parameters included carbamidomethylation of cysteine as a fixed modification; deamidation of asparagine and oxidation of methionine or tryptophan as common dynamic modifications; and rare dynamic modifications of *N*-terminal acetylation and formation of pyroglutamate from *N*-terminal glutamine or glutamate. Precursor and fragment ion mass tolerances were set to 7 and 10 ppm, respectively. The false discovery rate was estimated via a two-dimensional target decoy strategy [42] and adjusted to 1% (or 20 reverse count). An automatic cutoff for the Byonic® peptide score was chosen.

After initial data analysis via the default HCP workflow provided by Byos®, putative in silico peptides were added in Byologic® using the *Add missing* in silico *peptides* via *existing peptides* software component. In brief, this algorithm requires a collection of MS data files representing samples that were analyzed under identical chromatographic conditions, leading to sufficiently repeatable retention times (Electronic Supplementary Material (ESM) Fig. S1). For each peptide detected at least once via a fragment ion spectrum, the algorithm collects all retention times from all samples where this peptide was found on the MS/MS level. It then searches in the remaining samples on the full-scan MS level for signals at these retention times (with predefined tolerances for peak position and width), since such signals might represent peptides that did not trigger a fragmentation event. For each of these signals, the software calculates two measures to evaluate if the signal indeed originates from the respective peptide: (1) the deviation of the experimental mass from the theoretical peptide mass and (2) the so-called MS1 correlation, which is the Pearson correlation coefficient between the experimental isotope distribution and the isotope distribution of a theoretical peptide that has the same number of residues but consists entirely of averagine. (Averagine is a model for the “average” amino acid; its molecular formula is C_4.9384_H_7.7583_N_1.3577_O_1.4773_S_0.0417_, corresponding to an average molecular mass of 111.1254 Da [43].) The search for putative peptide signals on the full-scan MS level employed the following tolerance settings: a shift of the retention time of up to 0.5 min (parameter “FeatureCenterTolerance”), and a change in the peak width of up to 0.1 min (parameter “FeatureDurationTolerance”).

Data exported from Byologic® was further processed and visualized in R [44] using packages from the tidyverse [45] and Bioconductor [46]. In particular, any provisional in silico peptide was dismissed if either (1) its mass deviation was larger than the 2.5th or 97.5th percentile of the mass deviations of all precursor ions for which a fragment ion spectrum was available, or (2) its MS1 correlation coefficient was smaller than the 5th percentile of the coefficients of all precursor ions with a fragment ion spectrum (ESM Fig. S2). Moreover, the data analysis script ensured that peptides derived from keratins or “non-HCPs” (i.e., standard peptides, trypsin, protein A, DPs) were not erroneously used to identify HCPs.

### Data and code availability

Raw mass spectrometry data and Byonic search results are available from Zenodo (10.5281/zenodo.3778440). All input files and data analysis scripts used in this study are available as ZIP archive (see ESM).

## Results and discussion

### Direct HCP identification in commercial drug products

For our study, we assembled a panel consisting of five Fc domain comprising biopharmaceuticals, including two IgG1-type mAbs, that is, rituximab (MabThera®) and bevacizumab (Avastin®), as well as the Fc-fusion protein etanercept. With regard to the latter, two different Enbrel® production batches distributed in the USA and the European Union as well as the approved biosimilar Benepali® were analyzed. All of these therapeutic proteins originate from recombinant expression in CHO cells. Initially, we performed HCP identification in these commercial DPs via a *direct workflow* involving tryptic digestion of the respective DP, peptide analysis by RP-HPLC-MS/MS and protein identification against a CHO cell database supplemented with sequences of the respective DS, protein A, trypsin, and standard proteins (Fig. 1). With respect to the latter, we added *E. coli β*-galactosidase and bovine *β*-lactoglobulin to each DP prior to affinity chromatography, and spiked six standard peptides derived from *E. coli* ClpB into all samples prior to HPLC-MS measurements.

**Fig. 2:**
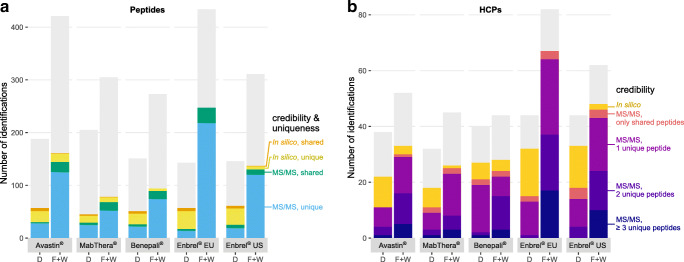
(**a**) Number of peptides and (**b**) number of proteins identified in the direct workflow (D) and in the combined flow-through and wash fractions of the depletion workflow (F+W) for each drug product. Each bar summarizes the results from three replicates (considering protein identification in one replicate sufficient). Colored bar sections highlight HCP-derived peptides and HCPs, respectively; moreover, they indicate credibility of detection and, in the case of peptides, their uniqueness. By contrast, light gray bars illustrate the total number of identified proteins or peptides, including putative contaminants (keratins) and “non-HCPs” (standard peptides, trypsin, protein A, drug products). “MS/MS” refers to identifications based on fragment ion spectra; “in silico” denotes identifications based solely on full-scan mass spectra. A peptide is considered “unique” if it matches a single sequence in the database; otherwise, it is classified as a “shared” peptide. Individual underlying peptide and HCP identifications are shown in ESM Fig. S3

In our initial data evaluation, we applied a conventional protein identification workflow: To identify a protein, at least one unique peptide with at least five residues had to be detected. A peptide is considered *unique* if it can only be assigned to a single protein in the sequence database used for identifying peptide spectra. In this way, we identified between 45 and 103 unique peptides (in Enbrel® EU and MabThera®, respectively). From these peptides, we inferred a number of corresponding database proteins, ranging from 16 to 23 (in MabThera® and Benepali®, respectively). Yet, a considerable fraction of these detections corresponded to putative contaminants (keratins) or “non-HCPs,” comprising spiked standard proteins, porcine trypsin from sample preparation, protein A bleeding from the affinity column, as well as the respective DSs. After elimination of these irrelevant protein identifications, a total number of 14 to 28 unique peptides remained (in Enbrel® EU and Avastin®, respectively; Fig. 2a, blue bar segments). Likewise, the number of detected HCPs decreased to between 9 and 19 (in MabThera® and Benepali®, respectively; Fig. 2b, violet bar segments).

### HCP identification upon drug substance depletion

**Fig. 3:**
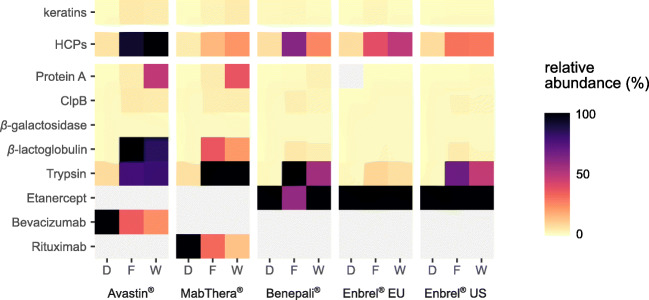
Relative protein quantification in the direct workflow (D) and in the flow-through (F) and wash (W) fractions of the depletion workflow for each drug product. Each value was obtained by (1) extracting and integrating precursor ion currents of up to three of the most intense (unique and shared) peptides per protein, (2) averaging these areas, (3) calculating the mean over three technical replicates, and (4) scaling to the maximum value in each column (M) so that all other values in this column are given as percent of the maximum. The two topmost rows show cumulative abundances of all keratins and HCPs, respectively

We next evaluated relative quantities of the identified HCPs with respect to the highly abundant DS. For this purpose, we considered the three most abundant peptides per protein and calculated cumulative abundances based on MS peak areas (Fig. 3). This analysis confirmed a vast excess of the respective therapeutic protein as compared with the identified HCPs. Co-elution of DS-derived peptides may thus compromise detection of HCP-derived peptides via ion suppression. To increase sensitivity for HCP detection, we aimed at enriching for HCPs by a depletion strategy: A solution containing 20 mg of the DS was applied to a protein A column to capture the biotherapeutic via its Fc domain (Fig. 1). Unbound HCPs, i.e., HCPs not interacting with the DS or the resin, were recovered in the flow-through. HCPs, bound non-specifically either to the protein A material or to the DS, on the other hand, were eluted from the loaded affinity column by applying a wash solution that disrupts their interactions with the DS or the resin. Since it was not necessary to keep the biotherapeutic in a functional state except its binding to protein A, we applied harsh wash conditions based on previous reports [39, 40, 47–49]: 10 mM Tris, 100 mM l*-*arginine, 1 M NaCl, and 500 mM TMAC, pH 10.0. The obtained flow-through and wash fractions were then analyzed by bottom-up RP-HPLC-MS/MS as described for the direct workflow.

Indeed, the depletion workflow (applying conventional data analysis) identified significantly more HCP-derived peptides and hence HCPs than the direct workflow. The increase in HCP identifications was most pronounced for Enbrel® EU, where the number of unique peptides and HCPs detected in at least one of the flow-through or wash fractions increased from 13 to 218 (Fig. 2a, blue bar segments) and 13 to 64 (Fig. 2b, violet bar segments), respectively. The substantial increase in the number of identifications upon depletion can be attributed to two factors: First, milligram amounts of the therapeutic protein loaded on the protein A column implicated comparatively high absolute amounts of HCPs. Second, DS capture prior to analysis prevented overloading of the RP-column with DS-derived peptides; thereby, it maintained separation performance, reduced overlapping elution of DS- and HCP-derived peptides, and notably lowered the dynamic range, as evident from an increase in relative HCP abundance (Fig. 3).

### Improved HCP identification via probabilistic protein inference and in silico peptides

Although the depletion workflow greatly improved the number of identified peptides and HCPs compared with the direct workflow, conventional data analysis still neglected valuable information present in the acquired spectra. Hence, we aimed at optimizing MS data evaluation by (a) employing a probabilistic protein inference algorithm that includes shared peptides, and (b) peptide detection on the full-scan MS level in the absence of MS/MS spectra.

Evidently, protein inference based on unique peptides will ignore any peptide detected by MS/MS if it matches more than one protein in the sequence database. Several algorithms have been proposed that include these so-called shared peptides when determining the protein content of a sample [50, 51]. Here, we relied on the probabilistic model implemented in Byonic™, which ranks protein identifications according to their total evidence in the peptide library and assigns each shared peptide to the highest-ranking protein [52]. Moreover, this model simultaneously controls both the peptide-to-spectrum match and protein false discovery rate via a two-dimensional target decoy strategy. Thereby, it warrants a large number of protein identifications while ensuring that both false discovery rates remain reasonably low [42]. When applied to our data from the depletion workflow, the probabilistic algorithm considered between ten and 29 shared peptides (in Enbrel® US and Enbrel® EU, respectively; Fig. 2a, green bar segments). These peptides enabled identification of one to three additional HCPs (in Avastin® and the Enbrel® batches, respectively; Fig. 2b, red bar segments).

Furthermore, we expanded peptide detection to the full-scan MS level, as low-abundant peptides may fail to trigger an MS/MS event. For this purpose, we assessed samples of each DP taking into account retention times, intact peptide masses, and isotope patterns [53, 54]. Accordingly, a peptide was identified in silico (i.e., in the absence of a fragment ion spectrum), and therefore denoted as “*in silico* peptide”, if it met the following three criteria: (1) The retention time of its intact peptide ion deviated at most 30 s from the retention time window spanned by all identical intact peptide ions identified via MS/MS in another sample of the same drug product. (2) The mass deviation of its intact peptide ion (as measured in ppm) fell within the 2.5th and 97.5th percentile of the mass deviations of all precursor ions for which a fragment ion spectrum was available (ESM Fig. S2a). (3) The experimental isotope pattern of its intact peptide ion adequately matched the theoretical averagine distribution (i.e., its Pearson correlation coefficient exceeded the 5th percentile of the coefficients of all precursor ions with a fragment ion spectrum) (ESM Fig. S2b). By re-assessing data from the depletion workflow in light of these criteria, we were able to detect up to 17 additional peptides (in Avastin®; Fig. 2a, yellow bar segments) and up to four additional HCPs (in Benepali®; Fig. 2b, yellow bar segments) that had been previously detected only in the direct workflow via MS/MS.

The different methods of identifying peptides and inferring proteins may even be used to classify detections on an ordinal scale of credibility (as indicated by the color scheme used in Fig. 2). One might consider HCP identifications supported by fragment ion spectra of several unique peptides to be more credible than those supported merely by in silico peptides, i.e., peptides identified only on full-scan MS level. Likewise, peptides identified via MS/MS are more credible than these in silico peptides.

### Comparative analysis of HCP profiles

In total, joint application of the depletion workflow and in-depth data analysis identified 127 distinct HCPs across all samples, based on 537 different peptides (ESM Fig. S3). Interestingly, although all investigated drugs were produced in CHO cells, only three HCPs were found in all of them, namely titin, nestin, and anionic trypsin-2 (ESM Fig. S4). These common HCPs were presumably co-purified with the therapeutic protein, probably by interacting with the IgG1-type Fc-subunit that is present in all investigated DSs, as previously suggested [37]. Moreover, several of the HCPs listed in ESM Fig. S3, e.g., clusterin, E3 ubiquitin-protein ligase, peroxiredoxin-1, glyceraldehyde-3-phosphate dehydrogenase, and 78-kDa glucose-regulated protein, have previously been identified in various DSs [26, 32, 37, 55, 56]. Thus, these HCPs may represent commonly occurring contaminants in CHO-based production systems. Several provisional HCP identifications (cationic trypsin-3, G3HUA1; anionic trypsin-2 fragment, G3HUC0; and Ig-like domain-containing protein, G3IMG2) were based solely upon peptides that could also be generated from non-HCPs via unspecific cleavage; hence, these proteins were removed from the final report. Nevertheless, other arguable HCP identifications were retained. For instance, identification of serum albumin (G3IAL6) may be attributed to carry over of bovine serum albumin that was used as standard protein in quality control runs. However, four of five peptides were unique for the hamster protein. Likewise, desmoplakin (G3HD94) and junction plakoglobin (G3HLU9) might be associated with contaminating keratins, but were nevertheless considered as HCPs, since they were identified only in a single drug product.

Although description of individual HCP identifications may provide basic insight into the HCP contents of the analyzed DPs, we aimed at an overall comparison of HCP profiles, i.e., the set of all HCPs identified in a given replicate, fraction, or DP (Fig. 1, bottom). To this end, HCPs and peptides of different samples were compared by calculating pairwise Jaccard indices, an elementary measure of set similarity: Applied to HCP profiles, a value of 1 indicates that exactly the same HCPs have been identified in two samples, while a value of 0 denotes two samples that do not share any HCPs. Computation of Jaccard indices facilitated the evaluation of (a) workflow repeatability (via comparison of HCP profiles on the replicate level), (b) co-purification tendency of HCPs during protein A chromatography (flow-through and wash fraction level), and (c) drug-to-drug versus batch-to-batch variability (DS level).

**Fig. 4:**
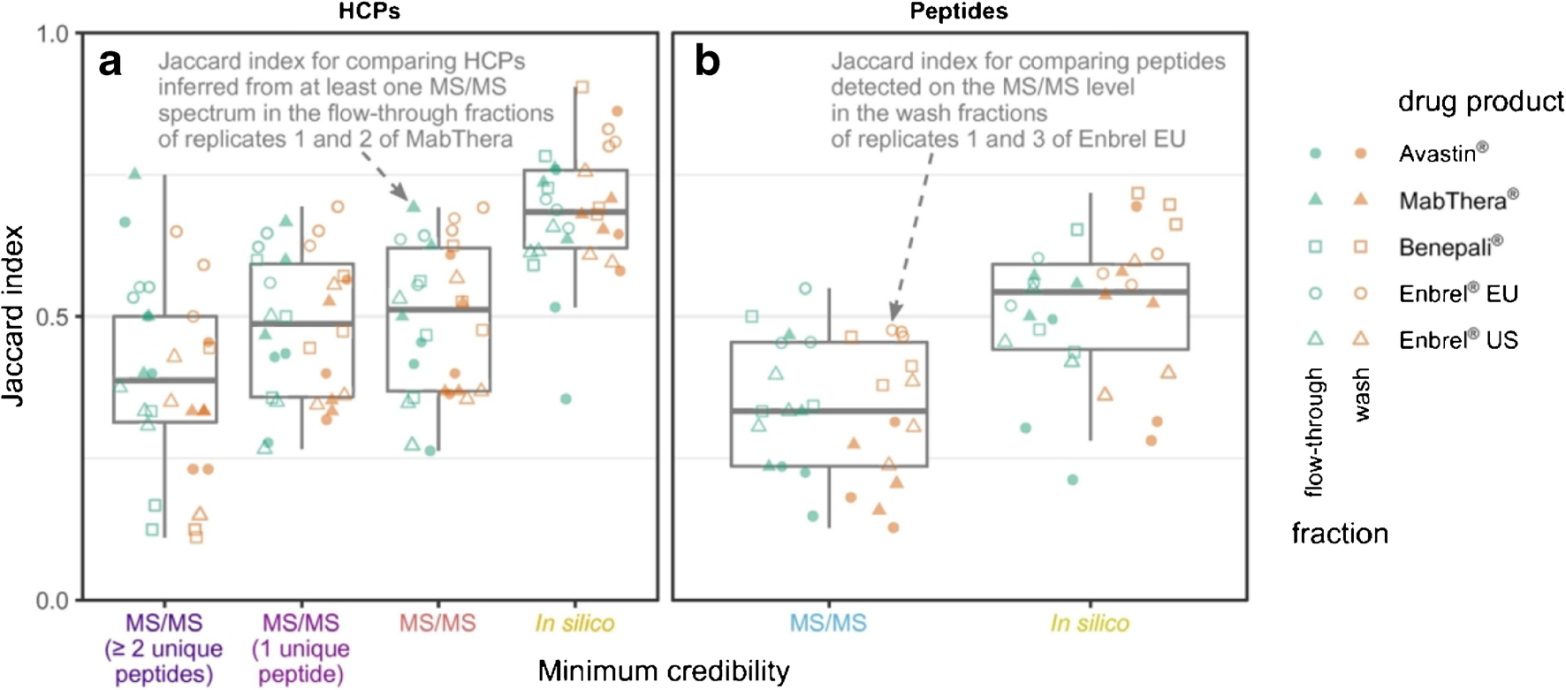
Repeatability of the depletion workflow depending on the minimum level of credibility required for identification of (**a**) HCPs or (**b**) HCP-derived peptides. Each point represents a comparison between two replicates of a fraction as quantified by the Jaccard index (two points have been annotated exemplarily). Boxes summarize all 30 comparisons for a given level of credibility (5 drug products × 2 fractions × 3 replicate combinations) via their quartiles, with whiskers extending to the smallest or largest value no further than 1.5 interquartile ranges away from the lower or upper quartile, respectively. Colors of *x*-axis tick labels correspond to the ones used in Fig. 2

First, to assess the repeatability of the depletion workflow, we prepared and analyzed three independent replicates and compared HCP profiles within the ten obtained fractions (i.e., flow-through and wash for five DPs). As expected, repeatability depended upon the minimum level of credibility that was accepted for a HCP to be identified (Fig. 4a): If only HCPs corroborated by MS/MS spectra of at least two unique peptides were taken into account, Jaccard indices merely assumed a low median value of 0.39. However, the repeatability increased if less credible identifications were included in the comparison, rising to a median value of 0.68 if HCP identifications based solely on in silico peptides were included. This improvement was accompanied by a decrease in variability as measured by the interquartile range. Taken together, these observations demonstrate that in silico peptides generally improve the repeatability of the depletion workflow. Comparable results were obtained for the repeatability of peptide identifications (Fig. 4b).

Second, to characterize the co-purification tendency of HCPs during protein A chromatography, HCP profiles of the flow-through and wash fractions were compared for each DP (ESM Fig. S5; the HCP profile of a fraction comprised all HCPs detected in at least one replicate of this fraction). Most HCPs were identified within both fractions, corresponding to Jaccard indices ranging from 0.75 to 0.91. This high degree of similarity suggests low-affinity interactions between the shared HCPs and the DS or the protein A resin, resulting in their partial retention [11, 38–40]. Yet, some HCPs occurred exclusively in the wash or flow-through, indicating high or no affinity, respectively. Wash-exclusive detections might flag HCPs that should be particularly monitored during the production process and extensively tested for any immunogenic effects: As these HCPs bind tightly to the DS, they are most likely to be co-purified with the therapeutic protein.

**Fig. 5:**
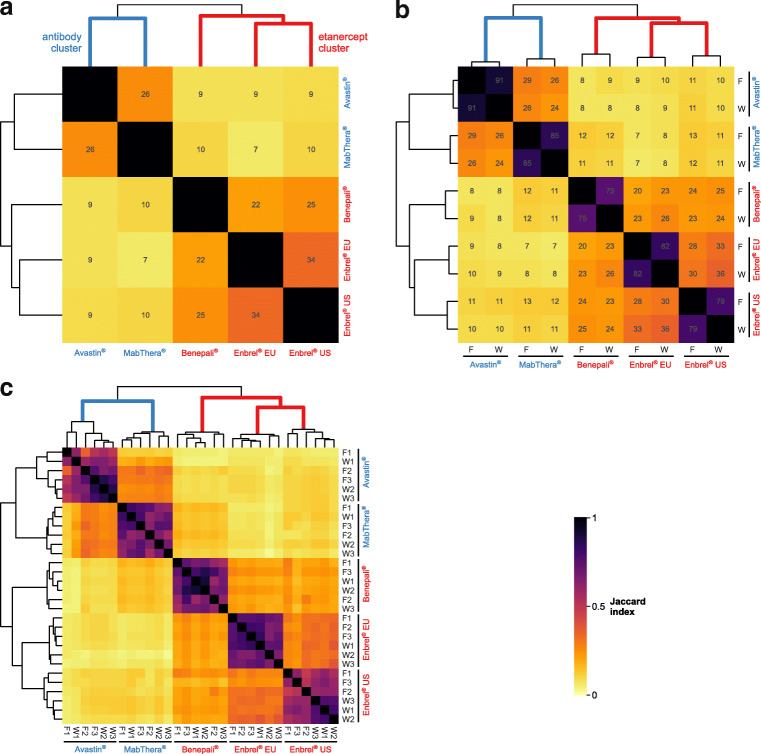
Comparison of HCP profiles on the level of (**a**) DPs, (**b**) flow-through (F) and wash (W) fractions from the depletion workflow, and (**c**) replicates 1 to 3 for these fractions. Heatmap colors correspond to Jaccard indices *J*, whose numerical values (in percent) appear in panels (**a**) and (**b**). Dendrograms and derived row and column orders result from hierarchical clustering employing Jaccard distances 1 − *J* as measure of dissimilarity. Calculation of Jaccard indices involved HCPs irrespective of credibility detection. The antibody and etanercept clusters are highlighted in blue and red, respectively

Third, to evaluate drug-to-drug and batch-to-batch variability of HCPs, HCP profiles of all DPs were compared via hierarchical clustering, using Jaccard distance (i.e., one minus the Jaccard index) as measure of dissimilarity (Fig. 5a; the HCP profile of a DP comprised all HCPs detected in at least one fraction of the depletion workflow). The corresponding dendrogram revealed two main clusters that contained the two antibodies and the three etanercept products, respectively. Within the etanercept cluster, a subcluster comprised the two Enbrel® batches, indicating a different HCP profile between Enbrel® and Benepali®. Remarkably, this overall cluster structure remained intact even when comparisons were made on the fraction or replicate level (Fig. 5b, c) or when using peptide profiles (ESM Fig. S6). Hence, hierarchical clustering was insensitive to random errors in the HCP repertoires, which were particularly pronounced on the level of replicates.

Overall, dendrograms suggested that the HCP content of a given DP depends on the structural properties of the respective DS, with similar structures implicating similar HCP profiles: The HPC profiles of the two mAbs are about as similar as the profiles of Benepali® and the Enbrel® cluster. Within a single structural class, different manufacturing processes result in distinct HCP profiles, as exemplified by etanercept: Here, the two Enbrel® batches are more similar to each other (comparable manufacturing process) than to Benepali® (biosimilar with different manufacturing process). Despite these observed tendencies, comprehensive analyses of a larger number of production batches will be required to compensate for batch-to-batch variations as well as inherent variability of the method.

## Conclusions

In this study, we explored different evaluation strategies of HPLC-MS/MS data for HCP discovery in highly pure DPs of biotherapeutics. Our data analysis protocol extended conventional approaches, i.e., HCP identification based on fragment ion mass spectra of unique peptides, by probabilistic protein inference and detection of peptides on the full-scan MS level. In conjunction with an experimental workflow involving DS depletion by protein A affinity chromatography and bottom-up proteomics via HPLC-MS/MS, this protocol significantly increased the number of identified peptides and HCPs. These detections allowed us to quantify similarities in HCP repertoires of several technical replicates and differently manufactured biotherapeutics, which may be used to evaluate workflow repeatability and drug-to-drug versus batch-to-batch variability, respectively. Taken together, these results underline the importance of utilizing the depth of information available from experimental HPLC-MS/MS data which is only partially used by standard data evaluation strategies. What is more, the demonstrated applicability of full-scan MS data for HCP identification purposes lays the basis for extended data evaluation strategies. For example, one might systematically determine accurate mass-retention time tags for all peptides from a given expression host, and then include this information in the algorithm for scoring full-scan MS and even MS/MS identifications [54].

Our findings demonstrate that the improved data analysis protocol is a flexible tool both for HCP discovery and for comparability studies of structurally diverse DPs based upon HCP profiles. If monitoring or absolute quantification of individual HCPs is required, results from our workflow may be directly used to devise targeted proteomics approaches based upon reaction monitoring techniques. Moreover, the protocol can easily be implemented into existing bottom-up setups based on HPLC-MS/MS. Notably, while we employed protein A affinity chromatography for DS depletion, our data analysis protocol is independent of this method and will readily work with novel techniques such as molecular weight cutoff enrichment [41]. The presented data evaluation workflow may thus be implemented at different stages of biopharmaceutical production, with comparative HCP profiles being especially attractive in the context of biosimilar development.

## Electronic supplementary material

ESM 1 (PDF 4231 kb).

ESM 2 (7Z 13101 kb).

## Abbreviations

CHO: Chinese hamster ovary
DP: Drug product
DS: Drug substance
ELISA: Enzyme-linked immunosorbent assay
HCP: Host cell protein
HPLC: High-performance liquid chromatography
mAb: Monoclonal antibody
MS: Mass spectrometry
MS/MS: Tandem mass spectrometry
