# Supplementary material for: Exploring sample preparation and data evaluation strategies for enhanced identification of host cell proteins in drug products of therapeutic antibodies and Fc-fusion proteins
Source: Anal Bioanal Chem. 2020 Jul 20;412(24):6583–93. doi: 10.1007/s00216-020-02796-1 (PMC7442769; doi:10.1007/s00216-020-02796-1)
Supplement: Supplementary file 1 — (PDF 4231 kb). [file 216_2020_2796_MOESM1_ESM.pdf]

Analytical and Bioanalytical Chemistry

Electronic Supplementary Material

Exploring sample preparation and data evaluation strategies for enhanced identification of host cell proteins in drug products of therapeutic antibodies and Fc-fusion proteins

Wolfgang Esser-Skala, Marius Segl, Therese Wohlschlager, Veronika Reisinger, Johann Holzmann, Christian G. Huber

Additional files available under “Supplementary material”.

Contents

Supplementary figures *3*
Figure S1 3
Figure S2 4
Figure S3 5
Figure S4 6
Figure S5 6
Figure S6 7

Supplementary files *8*
Supplementary file 1: `data.zip` 8

Code for data analysis and figures *9*
Preparation of datasets 9
Jaccard distance calculation 19
Figure 1: Workflow 22
Figure 2a: Peptide count 22
Figure 2b: HCP count 24
Figure 3: Protein abundances 26
Figure 4: Repeatability 28
Figure 5: HCP Jaccard plots 31
Other analyses 34

Code for supporting figures *42*
Figure S1: Retention time stability 42
Figure S2a: PPM distribution 43
Figure S2b: MS1 correlation distribution 44
Figure S3a: Peptide credibility 46
Figure S3b: HCP credibility 49
Figure S4: Distribution over drugs 52
Figure S5: Overlap flow-through/wash 54
Figure S6: Peptide Jaccard plots 58

Session info *62*

References *65*

Supplementary figures

Figure S1

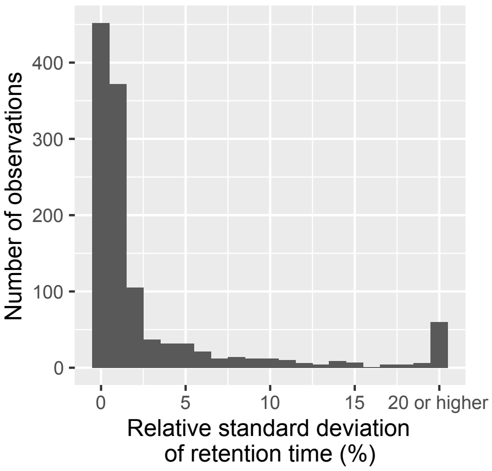

Chromatographic repeatability, as measured by relative standard deviations of peptide retention times. Each observation corresponds to the standard deviation of all retention times observed across all chromatographic runs for a given peptide with a given set of modifications, divided by the mean of these retention times.

Figure S2

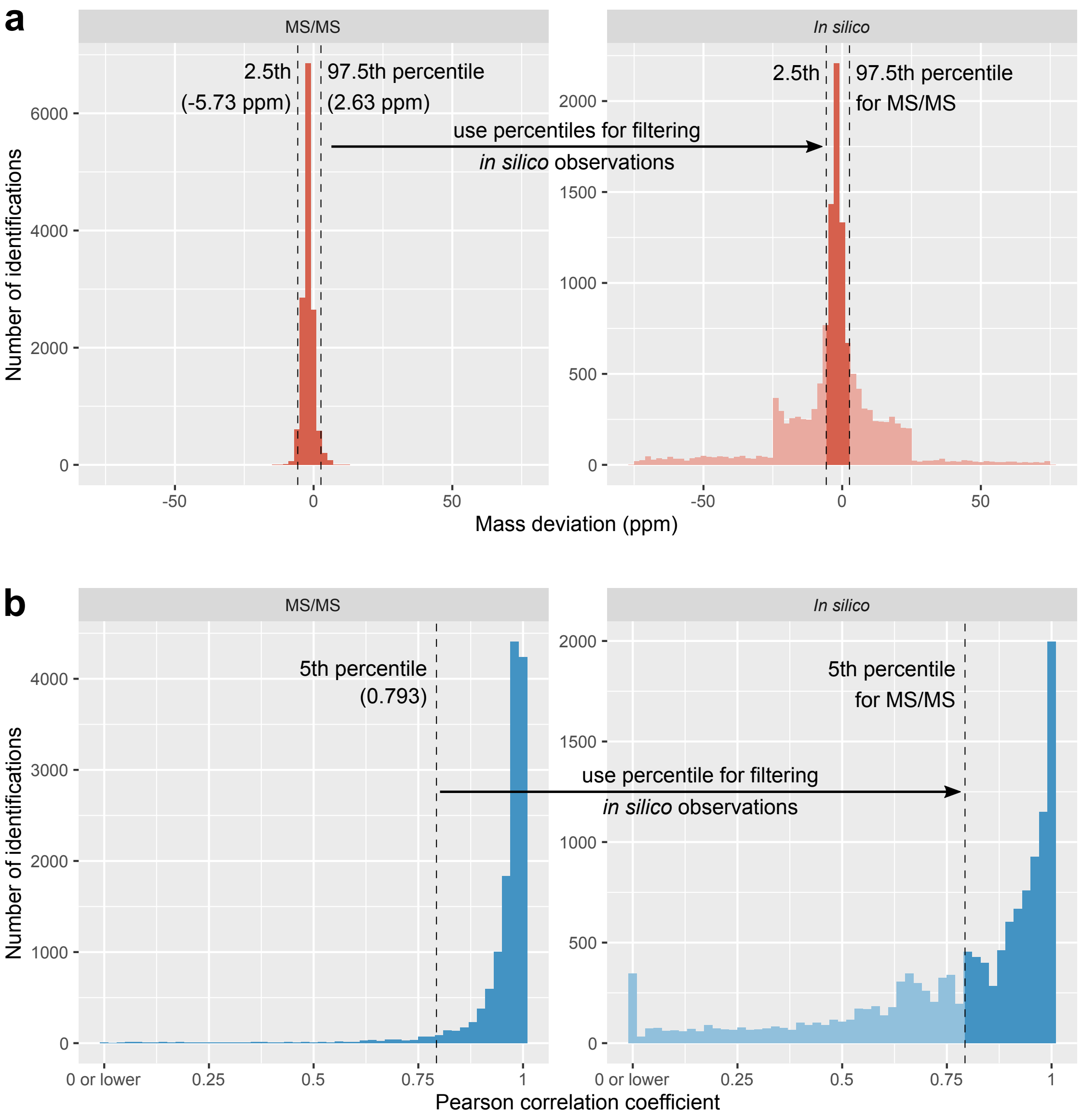

Distribution of (a) deviations from the theoretical mass and (b) Pearson correlation coefficients between the experimental isotope distribution and the theoretical averagine distribution for peptides detected *via* fragment ion spectra (MS/MS) and putative *in silico* peptides, respectively (*i.e.*, peptides identified on the full-scan MS level). Histograms were derived from all drug products, fractions, and replicates (27523 observations). Arrows indicate that percentiles calculated for MS/MS data were used for filtering *in silico* data.

Figure S3

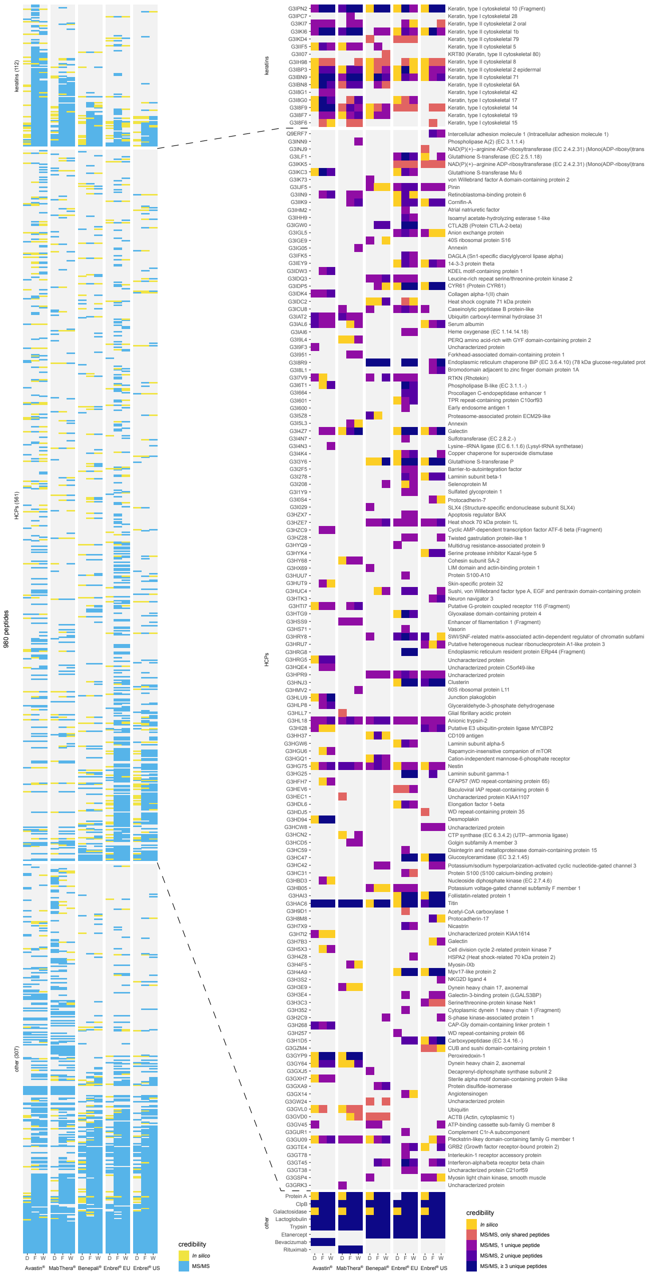

Peptides (left) and proteins (right) identified in the direct workflow (D) and in the flow-through (F) and wash (W) fractions of the depletion workflow for each drug product (vertical subdivisions). Horizontal subdivisions and dashed lines group observations into keratins, HCPs, and other proteins. Colors indicate credibility of detection. *(Zoom in to view details.)*

Figure S4

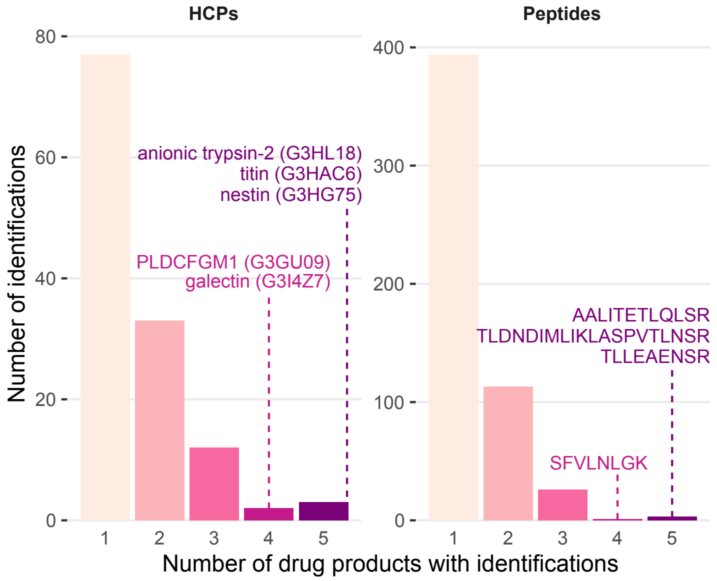

Number of HCPs and HCP-derived peptides identified across several drug products. Proteins and peptides shared by four or five drug products are quoted by name or sequence, respectively.

Figure S5

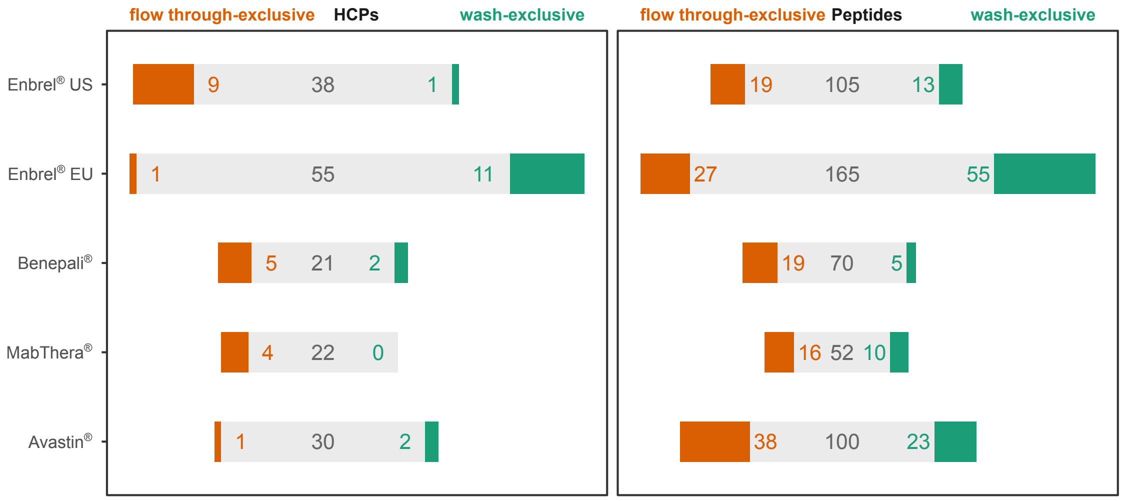

Number of HCPs and HCP-derived peptides that are either found in both fractions collected during the depletion workflow (gray), or only occur in the flow-through (brown) or wash (green) fraction, respectively.

Figure S6

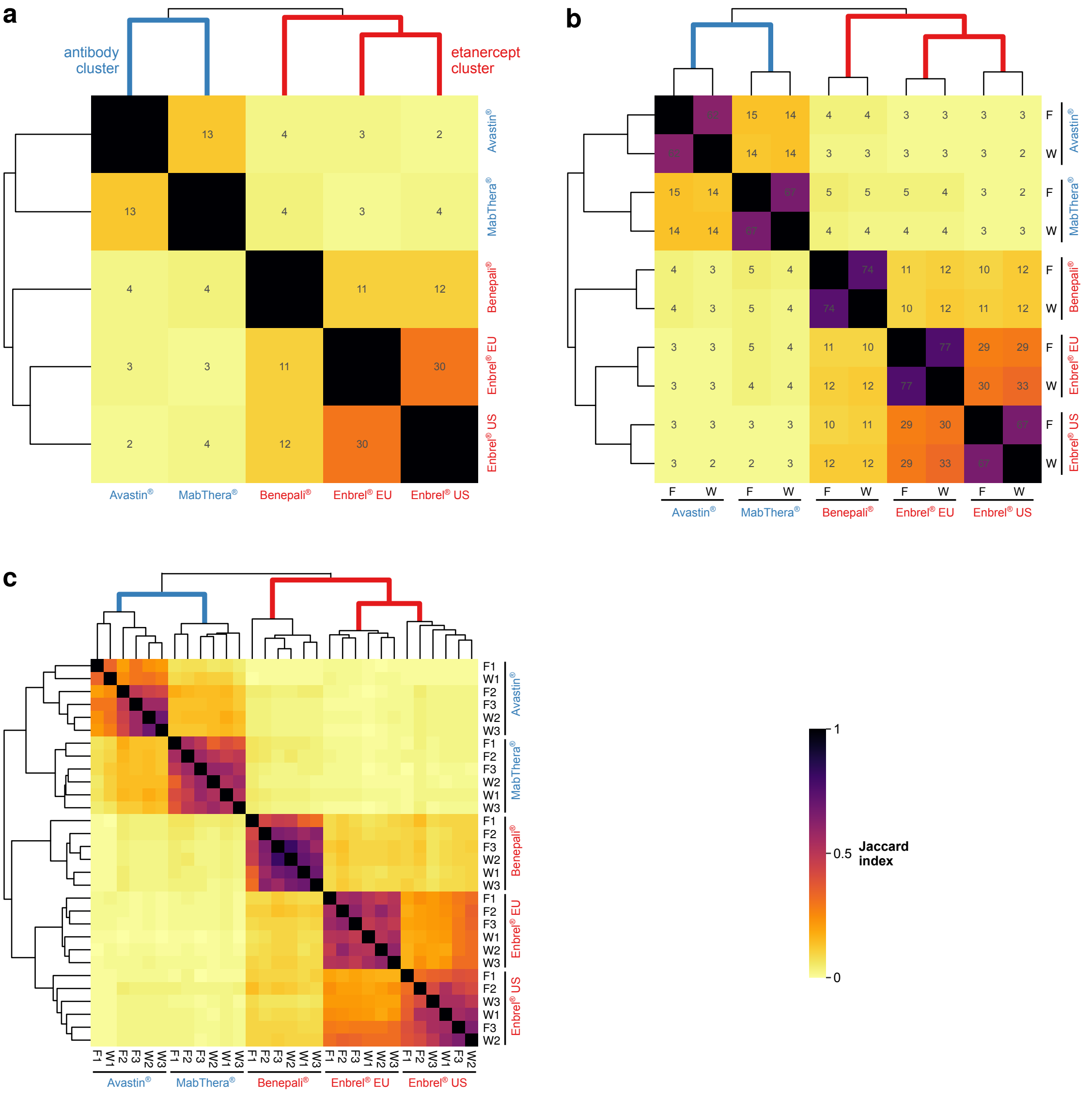

Comparison of HCP-derived peptide profiles on the level of (a) drug products, (b) flow-through (F) and wash (W) fractions from the depletion workflow, and (c) replicates 1 to 3 for these fractions. Heatmap colors correspond to Jaccard indices J, whose numerical values appear in heatmaps (a) and (b) in percent. Dendrograms and derived row and column orders result from hierarchical clustering employing Jaccard distances $1 - J$ as measure of dissimilarity. Calculation of Jaccard indices involved *in silico* peptides as well as those detected on the MS/MS level. The antibody and etanercept clusters are highlighted in blue and red, respectively.

Supplementary files

Supplementary file 1: `data.zip`

Files in this ZIP archive allow to reproduce all results presented in the manuscript. CSV files comply with RFC 4180[1], with a hash character in column 1 denoting comment lines.

The folder `data/` contains two subfolders:

- `byologic/` – Byologic® search results for the five investigated drug products
- `sequence_databases/` – databases in FASTA format containing Chinese Hamster Ovary cell protein sequences, supplemented with sequences of the respective drug substance, protein A, trypsin, *E. coli* ClpB and β-galactosidase, and bovine β-lactoglobulin

In addition, `supporting_information.Rmd` contains this document in R Markdown format[2]. Conversion to PDF *via* `knitr`[3] requires `citation-style.csl`, `orcid.pdf`, `references.bib`, `si_template.tex`, and all files in `supplementary_figures/`.

Code for data analysis and figures

Data analysis was conducted in R[4] using packages from the tidyverse[5] and Bioconductor[6]. Publication-quality figures were prepared with Inkscape v0.91 (`https://inkscape.org`) and GIMP v2.8.16 (`https://www.gimp.org`).

```
library(Biostrings)
library(tidyverse)
library(pheatmap)
library(viridis)
library(janitor)
library(fs)
library(httr)
```

Save generated figures in `./raw_figures/`. Create this folder if necessary.

```
dir.create(file.path(".", "raw_figures"), showWarnings = FALSE)
ggsave_default <- function(filename, ...) {
  ggsave(str_glue("raw_figures/{filename}.svg"), units = "cm", ...)
}
```

HCP credibility uses five colors from the colorblind-friendly Plasma color scale (`http://bids.github.io/colormap/`).

```
color_scale_credibility_hcp <- c(
  plasma(4, direction = -1, end = .9)[1:2],
  plasma(3, direction = -1, end = .3)
)
```

Preparation of datasets

Load data that was exported from Byologic®, ensuring that colums have the correct data type. Moreover, remove rows with a missing PPM value, since those observations correspond to incorrectly assigned *in silico* peptides.

```
peptide_data_raw <-
  dir_ls("data/byologic/", type = "file") %>%
  map_dfr(
    read_csv,
    col_types = cols(
      "Var. Pos.\r\nProtein" = "c",
      "_mod\r\nids" = "c",
      "Var. Pos.\r\nPeptide" = "c",
      "Start\r\nAA" = "c",
```

```
      "End\r\nAA" = "c",
      "Protein\r\nannotation" = "c",
      "Peptide Ranking" = "c"
    ),
    .id = "drug"
  ) %>%
  extract(drug, into = "drug", regex = "/([a-z_]*)\\.") %>%
  clean_names() %>%
  mutate(
    in_silico =
      in_silico %>%
      str_replace("Yes", "TRUE") %>%
      replace_na("FALSE") %>%
      parse_logical()
  ) %>%
  unite(samples, replicates, col = "sample", sep = "") %>%
  rename(protein = protein_accession) %>%
  {
    bind_rows(
      filter(., in_silico) %>%
        mutate(
          ms1_correlation = parse_double(ms1_correlation),
          ppm = parse_double(ppm)
        ),
      filter(., !in_silico) %>%
        mutate(
          ms1_correlation =
            ms1_correlation %>%
            str_split(";") %>%
            map_dbl(~.x %>% parse_double() %>% mean()),
          ppm =
            ppm %>%
            str_split(";") %>%
            map_dbl(~.x %>% parse_double() %>% mean())
        )
    )
  } %>%
  filter(!is.na(ppm))
```

Send a query to the UniProt `uploadlists` API in order to retrieve protein names associated with the identifiers in the Byologic® search results. This query yields the data frame `protein_data`.

```
uniprot_query_ids <-
  peptide_data_raw %>%
  extract(
    protein_name,
    into = "protein_id",
    regex = "\\|([:alnum:]+)\\|"
  ) %>%
  pull(protein_id) %>%
  unique() %>%
  str_c(collapse = " ")

uniprot_query_params <- list(
  from = "ACC+ID",
  to = "ACC",
  format = "tab",
  query = uniprot_query_ids,
  columns = "id,protein names"
)

uniprot_response <- POST(
  "https://www.uniprot.org/uploadlists/",
  query = uniprot_query_params
)

protein_data <-
  uniprot_response %>%
  content() %>%
  read_tsv() %>%
  select(entry = Entry, name = `Protein names`) %>%
  arrange(entry)
```

Vectors containing names of "non-HCPs", *i.e.*, the drug substances, trypsin, protein A, and standard proteins.

```
non_hcps_database <- c(
  "Rituximab",
  "Avastin",
  "Etanercept",
  "Trypsin",
  "Lactoglobulin",
  "Galactosidase",
  "ClpB",
  "ProteinA"
```

```
)

non_hcps <- c(
  "Rituximab",
  "Bevacizumab",
  "Etanercept",
  "Trypsin",
  "Lactoglobulin",
  "Galactosidase",
  "ClpB",
  "Protein A"
)
```

keratins contains UniProt identifiers of keratins, wich are putative contaminants. cho_sequences is a named character vector containing all protein sequences in the CHO cell database.

```
keratins <-
  protein_data %>%
  filter(str_detect(name, regex("keratin", ignore_case = TRUE))) %>%
  pull(entry)

cho_sequences <-
  dir_ls("data/sequence_databases/", type = "file") %>%
  readAAStringSet() %>%
  unique() %>%
  as.character() %>%
  set_names(function(x) str_match(x, "\\|([:alnum:]+)\\|")[,2])
```

Define several character vectors containing sequences of all uniqe peptides in the database (unique_peptides), all peptides derived from keratins (keratin_peptides), and all peptides associated with non-HCPs (non_hcp_peptides).

```
unique_peptides <-
  peptide_data_raw %>%
  pull(sequence_unformatted) %>%
  unique() %>%
  magrittr::extract(
    str_glue("([KR]{.})|(^{.})") %>%
      map(
        str_which,
        string = cho_sequences
      ) %>%
      map_lgl(~length(.x) == 1)
```

```
  )

keratin_peptides <-
  peptide_data_raw %>%
  pull(sequence_unformatted) %>%
  unique() %>%
  magrittr::extract(
    str_glue("([KR]{.})|(^{.})") %>%
      map(
        str_which,
        string = cho_sequences[keratins]
      ) %>%
      map_lgl(~length(.x) > 0)
  )

non_hcp_peptides <-
  peptide_data_raw %>%
  pull(sequence_unformatted) %>%
  unique() %>%
  magrittr::extract(
    str_glue("([KR]{.})|(^{.})") %>%
      map(
        str_which,
        string = cho_sequences[non_hcps_database]
      ) %>%
      map_lgl(~length(.x) > 0)
  ) %>%
  c("TLDNDIMLIK", "TIAAN", "NYLNWYQQK")
```

`recode_dp_names()` converts drug product names in the datasets to the final printed form (*i.e.*, uppercase with a registered trademark sign).

```
recode_dp_names <- function(df, drug_col = drug) {
  dp_mapping <- c(
    "Avastin®" = "avastin",
    "MabThera®" = "rituximab",
    "Benepali®" = "benepali",
    "Enbrel® EU" = "enbrel_eu",
    "Enbrel® US" = "enbrel_us"
  )

  suppressWarnings(
    df %>%
```

```
      mutate(
        {{drug_col}} :=
          factor({{drug_col}}) %>%
          fct_recode(!!!dp_mapping) %>%
          fct_relevel(names(dp_mapping))
      )
  )
}
```

In order to filter *in silico* peptides, determine the 5th percentile of MS1 correlation values (`insilico_limits$q5_ms1cor`) as well as the 2.5th and 97.5th percentile of PPM values (`insilico_limits$q2.5_ppm` and `insilico_limits$q97.5_ppm`, respectively) for peptides detected *via* fragment ion spectra. Subsequently, remove all putative *in silico* peptides from the dataset that do not fall within these percentiles. This operation yields the data frame `peptide_data`, which represents the final dataset of all detected peptides.

```
insilico_limits <-
  peptide_data_raw %>%
  filter(!in_silico) %>%
  summarise(
    q5_ms1cor = quantile(ms1_correlation, .05),
    q2.5_ppm = quantile(ppm, .025),
    q97.5_ppm = quantile(ppm, .975)
  ) %>%
  as.list()

peptide_data <-
  peptide_data_raw %>%
  filter(
    !in_silico |
      (
        in_silico &
          ms1_correlation >= insilico_limits$q5_ms1cor &
          ppm %>% between(
            insilico_limits$q2.5_ppm,
            insilico_limits$q97.5_ppm
            )
      )
  ) %>%
  recode_dp_names() %>%
  mutate(
    protein =
      str_replace_all(
        protein,
```

```
        c("ProteinA" = "Protein A",
          "Avastin" = "Bevacizumab",
          "G3HUC0" = "Trypsin",
          "G3HUA1" = "Trypsin",
          "G3IMG2" = "Bevacizumab")
      )
  )
```

`peptide_abundances` collectes the following properties for each peptide on the level of replicates: Total XIC area, credibility (*i.e.*, detection *via* MS/MS or *in silico*), relative abundance, and type (HCP, keratin, or other). Only peptides with at least five residues will be considered for calculating these properties.

```
peptide_abundances <-
  peptide_data %>%
  filter(peptide_length >= 5) %>%
  group_by(drug, sample, sequence_unformatted) %>%
  summarise(
    xic = sum(xic_area_summed, na.rm = TRUE),
    credibility = !all(in_silico)
  ) %>%
  mutate(
    rel_abundance = xic / max(xic) * 100,
    credibility = case_when(
      credibility ~ "MS/MS",
      TRUE        ~ "In silico"
    ) %>%
      as.ordered()
  ) %>%
  ungroup() %>%
  rename(sequence = sequence_unformatted) %>%
  mutate(
    type = case_when(
      sequence %in% keratin_peptides ~ "keratins",
      sequence %in% non_hcp_peptides ~ "other",
      TRUE                           ~ "HCPs"
    ) %>%
      as_factor() %>%
      fct_relevel("keratins", "HCPs")
  )
```

Below, the three properties credibility, relative abundance, and type will be aggregated at the fraction or workflow level. To this end, the function `aggregate_properties()` provides a common sequence of transformations.

```
aggregate_properties <- function(df, ...) {
  df %>%
  group_by(...) %>%
  summarise(
    credibility = max(credibility),
    rel_abundance = mean(rel_abundance),
    type = first(type)
  ) %>%
  mutate(rel_abundance = rel_abundance / max(rel_abundance) * 100) %>%
  ungroup()
}
```

peptide_abundances_fractions aggregates the properties of each peptide at the fraction level.

```
peptide_abundances_fractions <-
  peptide_abundances %>%
  mutate(sample = str_sub(sample, 1, 1)) %>%
  aggregate_properties(drug, sample, sequence)
```

Likewise, peptide_abundances_workflow aggregates these properties at the workflow level.

```
peptide_abundances_workflow <-
  peptide_abundances %>%
  mutate(sample = case_when(
    str_starts(sample, "D") ~ "D",
    TRUE                    ~ "F+W"
  )) %>%
  aggregate_properties(drug, sample, sequence)
```

Assemble data on identified HCPs, which will require two auxiliary functions. First, add_peptide_rank() ranks each peptide according to its abundance (that is, total XIC area of all observed charge states).

```
add_peptide_rank <- function(df) {
  df_rank <-
    df %>%
    group_by(
      drug, protein, sample, sequence_unformatted, mod_summary
    ) %>%
    summarise(xic = sum(xic_area_summed, na.rm = TRUE)) %>%
    group_by(drug, protein, sample) %>%
    mutate(peptide_rank = row_number(desc(xic))) %>%
```

```r
    ungroup() %>%
    select(-xic)

  df %>%
    left_join(
      df_rank,
      by = c("drug", "sequence_unformatted",
             "mod_summary", "protein", "sample")
    )
}
```

Second, `add_n_unique_peptides()` determines the number of unique peptides observed at the MS/MS level for each HCP.

```r
add_n_unique_peptides <- function(df) {
  n_unique_peptides <-
    df %>%
    filter(!in_silico, sequence_unformatted %in% unique_peptides) %>%
    group_by(drug, sample, protein) %>%
    summarise(n_peptides = n_distinct(sequence_unformatted)) %>%
    ungroup()

  df %>%
    left_join(n_unique_peptides, by = c("drug", "sample", "protein"))
}
```

`hcp_abundances` collects the following data for each identified protein on the level of replicates: Total XIC area, credibility, relative abundance, and type. Only the three highest-ranking peptides with at least five residues will be considered for calculating these properties.

```r
hcp_abundances <-
  peptide_data %>%
  filter(peptide_length >= 5) %>%
  add_peptide_rank() %>%
  add_n_unique_peptides() %>%
  mutate(
    credibility = case_when(
      !in_silico & n_peptides >= 3 ~ "MS/MS,   3 unique peptides",
      !in_silico & n_peptides == 2 ~ "MS/MS, 2 unique peptides",
      !in_silico & n_peptides == 1 ~ "MS/MS, 1 unique peptide",
      !in_silico                   ~ "MS/MS",
      TRUE                         ~ "In silico"
    ) %>%
```

```
      as.ordered()
  ) %>%
  group_by(drug, sample, protein) %>%
  summarise(
    xic = sum(xic_area_summed[peptide_rank <= 3], na.rm = TRUE) /
      n_distinct(peptide_rank[peptide_rank <= 3]),
    credibility = max(credibility)
  ) %>%
  mutate(rel_abundance = xic / max(xic) * 100) %>%
  ungroup() %>%
  mutate(
    type = case_when(
      protein %in% keratins ~ "keratins",
      protein %in% non_hcps ~ "other",
      TRUE                  ~ "HCPs"
    ) %>%
      as_factor() %>%
      fct_relevel("keratins", "HCPs")
  )
```

As above, aggregate HCP abundances on the level of fractions …

```
hcp_abundances_fractions <-
  hcp_abundances %>%
  mutate(sample = str_sub(sample, 1, 1)) %>%
  aggregate_properties(drug, sample, protein)
```

… and workflows.

```
hcp_abundances_workflow <-
  hcp_abundances %>%
  mutate(sample = case_when(
    str_starts(sample, "D") ~ "D",
    TRUE                    ~ "F+W"
  )) %>%
  aggregate_properties(drug, sample, protein)
```

In addition, protein abundances on the fraction level will be analyzed in a "compressed" form, where the abundances of all HCPs will be accumulated.

```
hcp_abundances_fractions_compressed <-
  hcp_abundances_fractions %>%
  filter(type == "other") %>%
  bind_rows(
```

```
    hcp_abundances_fractions %>%
      group_by(drug, sample, type) %>%
      summarise(
        credibility = max(credibility),
        rel_abundance = sum(rel_abundance)
      ) %>%
      mutate(protein = as.character(type)) %>%
      filter(protein != "other")
  ) %>%
  group_by(drug, sample) %>%
  mutate(rel_abundance = rel_abundance / max(rel_abundance) * 100) %>%
  ungroup()
```

Jaccard distance calculation

Define several functions that allow to compare HCP profiles by means of their Jaccard distance. `jaccard_matrix()` assembles a matrix with Jaccard indices. It requires a data frame with one column containing sample names (`sample_col`) and one column listing members of this sample (`member_col`, *e.g.*, protein identifiers).

```
jaccard_matrix <- function(df,
                           sample_col = sample,
                           member_col = member,
                           as_matrix = TRUE) {
  result <-
    df %>%
    pull({{sample_col}}) %>%
    unique() %>%
    list(x = ., y = .) %>%
    cross_df() %>%
    pmap_dfr(
      function(x, y) {
        ids_x <-
          df %>%
          filter({{sample_col}} == {{x}}) %>%
          pull({{member_col}})
        ids_y <-
          df %>%
          filter({{sample_col}} == {{y}}) %>%
          pull({{member_col}})
        tibble(
          x = x,
          y = y,
```

```
          members_x = list(ids_x),
          members_y = list(ids_y),
          size_intersection = length(intersect(ids_x, ids_y)),
          size_union = length(union(ids_x, ids_y)),
          index = size_intersection / size_union
        )
      }
    )

  if (as_matrix) {
    result %>%
      select(x, y, index) %>%
      pivot_wider(names_from = y, values_from = index) %>%
      column_to_rownames("x") %>%
      as.matrix()
  } else {
    result
  }
}
```

reorder_hclust_leaves() and reorder_hclust() are auxiliary functions that reorder dendrograms according to a string vector.

```
reorder_hclust_leaves <- function(dend, mat, ...) {
  wts <-
    c(...) %>%
    map(~str_which(rownames(mat), .)) %>%
    simplify() %>%
    order()
  reorder(dend, wts, min)
}

reorder_hclust <- function(mat, ...) {
  mat %>%
    dist() %>%
    hclust() %>%
    as.dendrogram() %>%
    reorder_hclust_leaves(mat, ...) %>%
    as.hclust()
}
```

jaccard_heatmap() draws a heatmap representing a matrix of Jaccard indices. Cells of this heatmap may have one of three sizes, in order to generate heatmaps with equal areas for drugs, fractions, and replicates.

```r
jaccard_heatmap <- function(mat, size = c("small", "medium", "large")) {
  size <- match.arg(size)
  cell_size <- 7.79 *
    case_when(
      size == "small"  ~ 1,
      size == "medium" ~ 3,
      TRUE             ~ 6
    )

  if (size == "small")
    display_numbers <- FALSE
  else
    display_numbers <-
      (mat * 100) %>%
      replace(near(., 100), NA) %>%
      format(digits = 0) %>%
      str_replace("NA", "") %>%
      str_trim() %>%
      matrix(nrow = nrow(mat))

  dend <-
    mat %>%
    reorder_hclust("Avastin", "MabThera",
                   "Benepali", "Enbrel EU", "Enbrel US")

  opts <-
    list(
      border_color = NA,
      breaks = seq(0, 1, length.out = 101),
      cellheight = cell_size,
      cellwidth = cell_size,
      cluster_cols = dend,
      cluster_rows = dend,
      color = rev(inferno(100)),
      display_numbers = display_numbers,
      fontsize = 8,
      legend = TRUE,
      number_format = "%.f"
    )

  p <- exec(pheatmap, mat, !!!opts)
  set_last_plot(p)
  invisible(p)
```

```
}
```

Figure 1: Workflow

This figure was drawn in Inkscape.

Figure 2a: Peptide count

`count_peptides()` reports the number of peptides in a dataset by drug, sample, type, credibility, and uniqueness.

```
count_peptides <- function(df) {
  df %>%
    mutate(
      uniqueness = case_when(
        sequence %in% unique_peptides ~ "unique",
        TRUE                          ~ "shared"
      ),
    ) %>%
    group_by(drug, sample) %>%
    count(type, credibility, uniqueness) %>%
    ungroup() %>%
    complete(
      drug, sample, type, credibility, uniqueness,
      fill = list(n = 0)
    )
}
```

Draw a bar chart showing the number of peptides for each drug and color the bars by credibility and uniqueness. Use selected colors (that is, numbers 2, 5, 4, and 3) from the Okabe-Ito color scale[7]. In addition, show gray bars in the background indicating the total number of peptides.

```
draw_peptide_count <- function(df) {
  total_abundances <-
    df %>%
    group_by(drug, sample) %>%
    summarise(n = sum(n))

  df %>%
    filter(type == "HCPs") %>%
    unite(credibility, uniqueness, col = "rel_uni") %>%
    ggplot(aes(sample, n)) +
    geom_col(data = total_abundances, fill = "grey92") +
```

```r
    geom_col(aes(fill = rel_uni)) +
    scale_x_discrete(name = "") +
    scale_y_continuous(
      name = "Number of identifications",
      expand = expansion(mult = c(0, .05))
    ) +
    scale_fill_manual(
      name = "credibility",
      values = c("#e69f00", "#f0e442", "#009e73", "#56b4e9")
    ) +
    facet_wrap(vars(drug), nrow = 1, strip.position = "bottom") +
    theme_bw() +
    theme(
      panel.border = element_blank(),
      panel.grid.major.x = element_blank(),
      panel.grid.minor = element_blank(),
      strip.background = element_rect(color = NA)
    )
}

peptide_abundances_workflow %>%
  count_peptides() %>%
  draw_peptide_count()
```

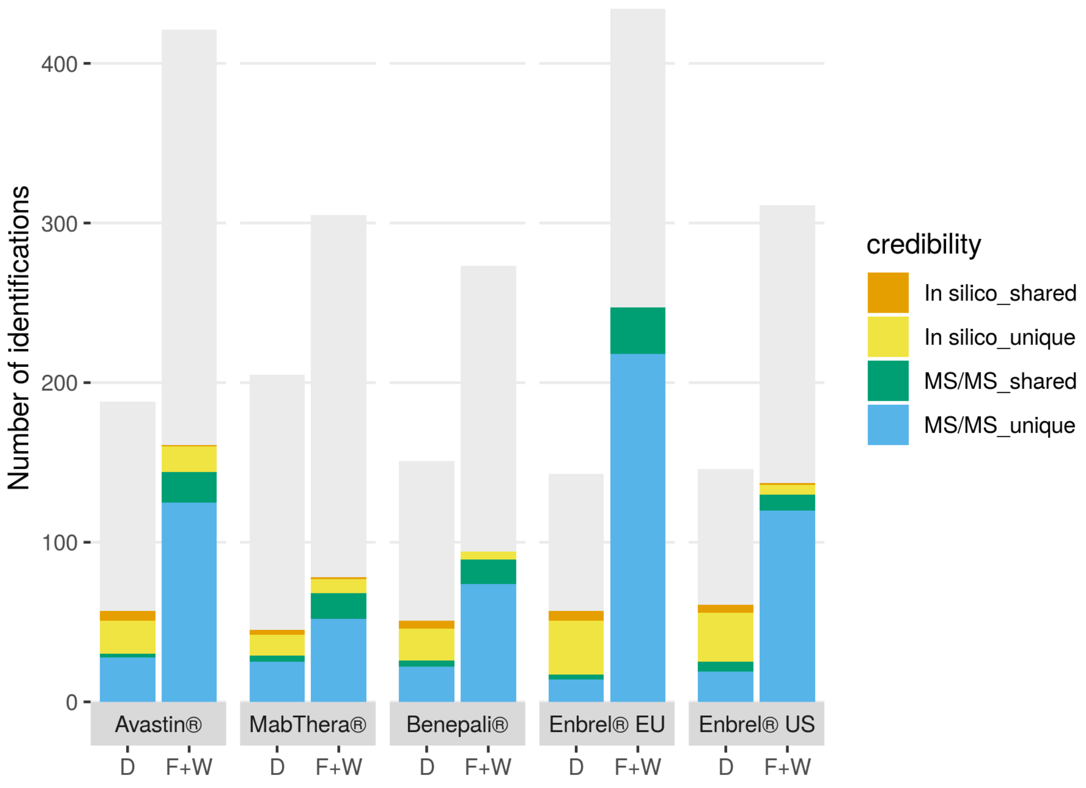


```
ggsave_default("figure_2a", width = 15.4, height = 12)
```

Figure 2b: HCP count

`count_hcps()` reports the number of proteins in a dataset by drug, sample, type, and credibility.

```
count_hcps <- function(df) {
  df %>%
    group_by(drug, sample, type, credibility) %>%
    summarise(n = n_distinct(protein)) %>%
    ungroup() %>%
    complete(drug, sample, type, credibility, fill = list(n = 0))
}
```

Draw a bar chart showing the number of HCPs for each drug and color the bars by credibility. In addition, show gray bars in the background indicating the total number of proteins.

```r
draw_hcp_count <- function(df) {
  total_abundances <-
    df %>%
    group_by(drug, sample) %>%
    summarise(n = sum(n))

  df %>%
    filter(type == "HCPs") %>%
    ggplot(aes(sample, n)) +
    geom_col(data = total_abundances, fill = "grey92") +
    geom_col(aes(fill = credibility)) +
    scale_x_discrete(name = "") +
    scale_y_continuous(
      name = "Number of identifications",
      expand = expansion(mult = c(0, .05))
    ) +
    scale_fill_manual(
      name = "credibility",
      values = color_scale_credibility_hcp,
      drop = FALSE,
    ) +
    facet_wrap(vars(drug), nrow = 1, strip.position = "bottom") +
    theme_bw() +
    theme(
      panel.border = element_blank(),
      panel.grid.major.x = element_blank(),
      panel.grid.minor = element_blank(),
      strip.background = element_rect(color = NA)
    )
}

hcp_abundances_workflow %>%
  count_hcps() %>%
  draw_hcp_count()
```

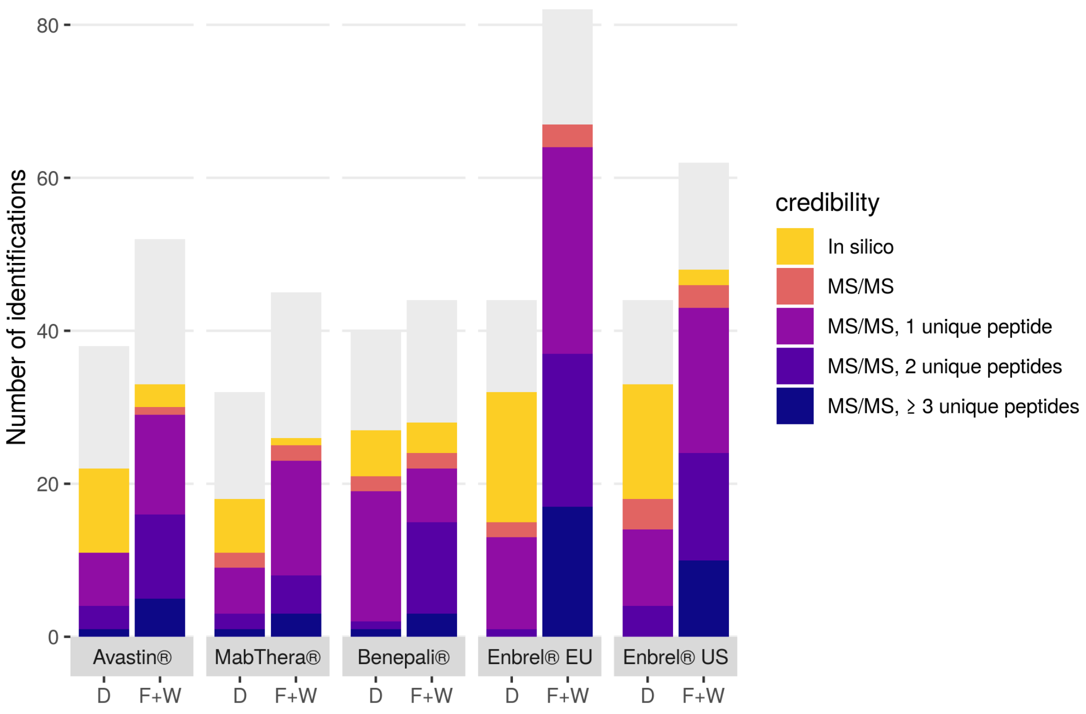


```
ggsave_default("figure_2b", width = 17, height = 12)
```

Figure 3: Protein abundances

Draw a heatmap showing estimated protein abundances.

```
draw_compressed_heatmap <- function(df) {
  y_order <- c(non_hcps, "HCPs", "keratins")

  df %>%
    mutate(
      protein =
        protein %>%
        as_factor() %>%
        fct_relevel(y_order) %>%
        as.numeric()
    ) %>%
    ggplot(aes(sample, protein)) +
    geom_tile(aes(fill = rel_abundance)) +
    scale_x_discrete(name = "", expand = expansion()) +
```

```
    scale_y_continuous(
      name = "",
      breaks = seq_along(y_order),
      labels = y_order,
      expand = expansion()
    ) +
    scale_fill_viridis(
      name = "relative abundance (%)",
      direction = -1,
      option = "A",
    ) +
    facet_grid(
      vars(type),
      vars(drug),
      scales = "free_y",
      space = "free_y",
      switch = "both"
    ) +
    theme_bw() +
    theme(
      axis.ticks.y.right = element_blank(),
      panel.border = element_blank(),
      panel.background = element_rect(fill = "#f2f2f2"),
      panel.grid = element_blank(),
      panel.spacing.x = unit(2, "mm"),
      strip.background = element_blank(),
      strip.placement = "outside",
      strip.switch.pad.wrap = unit(0, "mm"),
      strip.text.y = element_blank()
    )
}

hcp_abundances_fractions_compressed %>%
  draw_compressed_heatmap()
```

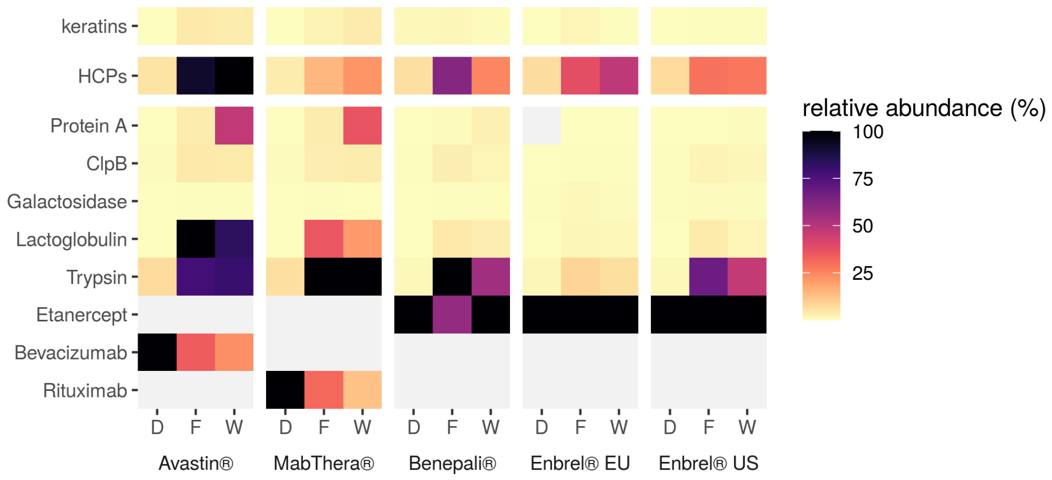


```
ggsave_default("figure_3", width = 17.9, height = 8.5)
```

Figure 4: Repeatability

`get_repeatability()` collects Jaccard indices obtained by pairwise comparison of all replicates for a fraction of a drug.

```
get_repeatability <- function(df, member_col) {
  result <-
    df %>%
    mutate(fraction = str_sub(sample, 1, 1)) %>%
    expand(drug, fraction, credibility) %>%
    pmap_dfr(
      function(drug, fraction, credibility) {
        df <-
          df %>%
          filter(
            type == "HCPs",
            drug == {{drug}},
            sample %>% str_starts(fraction),
            credibility >= {{credibility}}
          ) %>%
          unite(drug, sample, col = "sample")

        if (nrow(df) == 0)
          return(tibble())

        df %>%
```

```
          jaccard_matrix(member_col = {{member_col}}) %>%
          magrittr::inset(lower.tri(., diag = TRUE), NA) %>%
          as_tibble(rownames = "sample_1") %>%
          pivot_longer(
            -sample_1,
            names_to = "sample_2",
            values_to = "jaccard_index"
          ) %>%
          drop_na(jaccard_index) %>%
          mutate(min_credibility = {{credibility}})
      }
    )

  result %>%
    complete(
      nesting(sample_1, sample_2),
      min_credibility,
      fill = list(jaccard_index = 0)
    ) %>%
    extract(
      sample_1,
      into = c("drug", "fraction"),
      regex = "(.+)_(.)",
      remove = FALSE
    )
}

hcp_repeatability <-
  hcp_abundances %>%
  get_repeatability(protein)

peptide_repeatability <-
  peptide_abundances %>%
  get_repeatability(sequence)
```

Draw a boxplot summarizing these Jaccard indices. Use ColorBrewer's Dark2 color scheme for fractions[8].

```
bind_rows(
  "HCPs" = hcp_repeatability,
  "Peptides" = peptide_repeatability,
  .id = "level"
) %>%
```

```
mutate(
  min_credibility = ordered(min_credibility) %>% fct_rev(),
  fraction = case_when(
    fraction == "F" ~ "flow-through",
    TRUE            ~ "wash"
  )
) %>%
recode_dp_names() %>%
filter(
  !str_detect(sample_1, "_D"),
  min_credibility >= "MS/MS, 2 unique peptides"
  ) %>%
ggplot(
  aes(
    fct_recode(
      min_credibility,
      "MS/MS\n(1 unique\npeptide)" = "MS/MS, 1 unique peptide",
      "MS/MS\n( 2 unique\npeptides)" = "MS/MS, 2 unique peptides"
    ),
    jaccard_index
  )
) +
geom_boxplot(
  color = "gray50",
  outlier.alpha = 0
) +
geom_point(
  aes(color = fraction, shape = drug, group = fraction),
  position = position_jitterdodge(jitter.width = 1, seed = 42),
  alpha = .5
) +
scale_y_continuous(
  name = "Jaccard index",
  limits = c(0, 1),
  breaks = c(0, .5, 1),
  expand = c(0, 0)
) +
scale_color_brewer(
  palette = "Dark2",
  guide = guide_legend(override.aes = list(shape = 15, size = 6))
) +
scale_shape_manual(
  name = "drug product",
```

```
    values = c(16, 17, 0, 1, 2),
    guide = guide_legend(override.aes = list(alpha = 1))
  ) +
  facet_wrap(vars(level), scales = "free_x") +
  xlab("Minimum credibility") +
  theme_bw() +
  theme(
    panel.grid.major.x = element_blank(),
    strip.background = element_blank(),
    strip.text = element_text(face = "bold")
  )
```

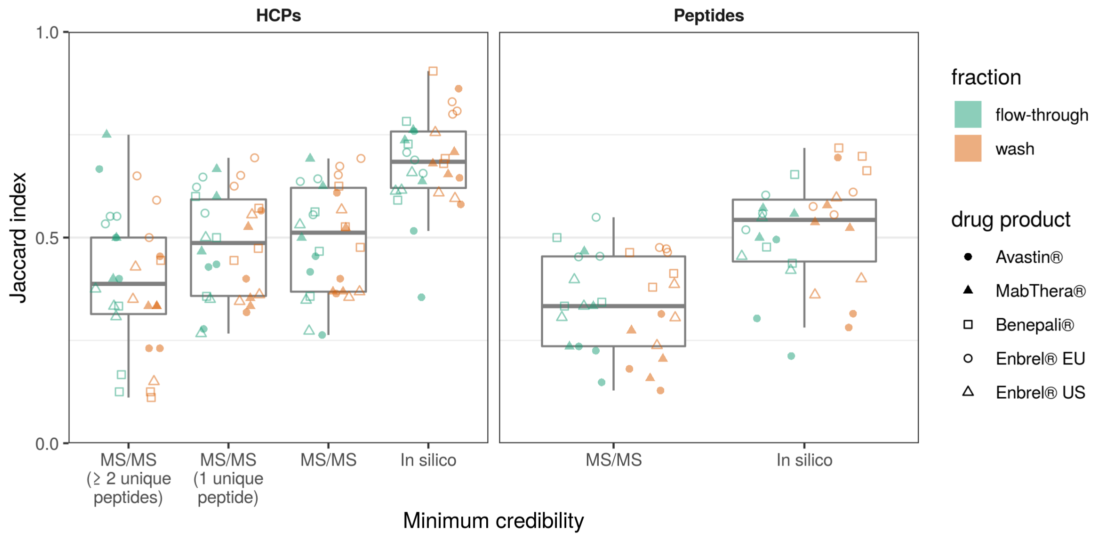


```
ggsave_default("figure_4", width = 20, height = 10)
```

Figure 5: HCP Jaccard plots

Draw heatmaps for comparison of HCP profiles on the level of drugs, …

```
jaccard_data_hcp <-
  hcp_abundances %>%
  filter(
    type == "HCPs",
    !str_starts(sample, "D"),
  )

jaccard_data_hcp %>%
```

```r
  jaccard_matrix(sample_col = drug, member_col = protein) %>%
  jaccard_heatmap(size = "large")
```

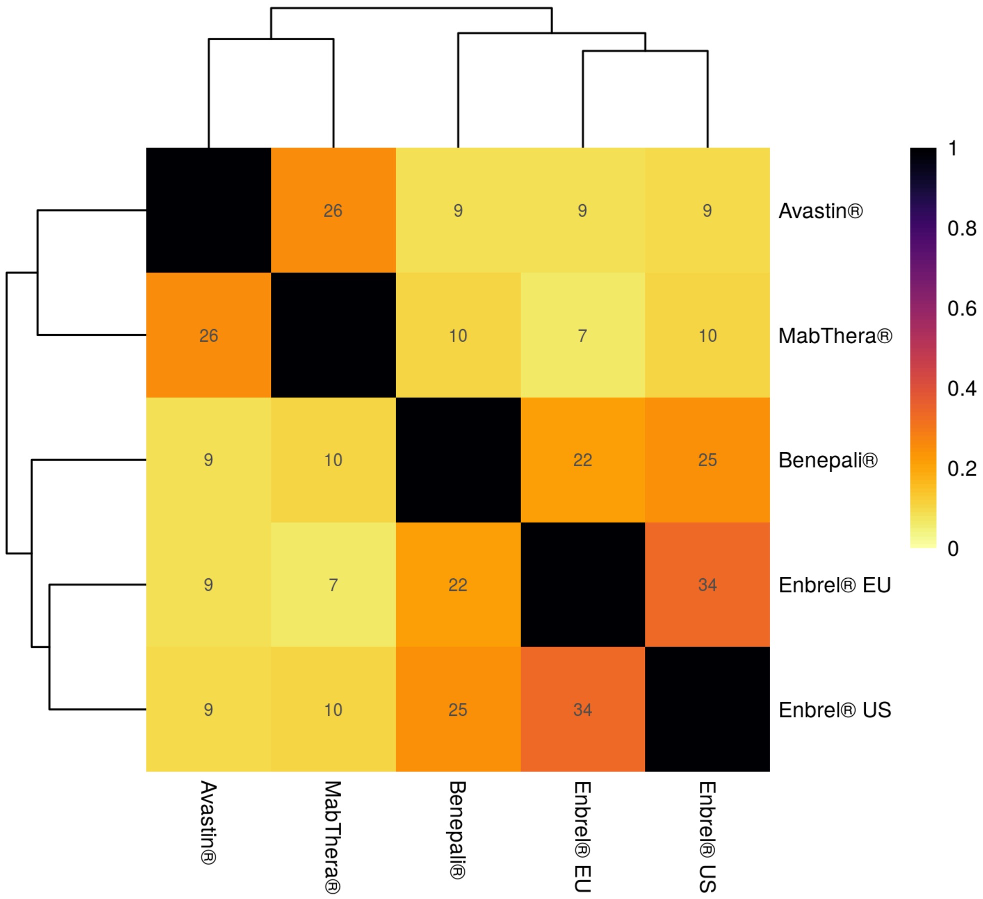


```r
ggsave_default("figure_5a", width = 13.6, height = 12.4)
```

... fractions, ...

```r
jaccard_data_hcp %>%
  mutate(sample = str_c(drug, str_sub(sample, 1, 1), sep = "_")) %>%
  jaccard_matrix(member_col = protein) %>%
  jaccard_heatmap(size = "medium")
```

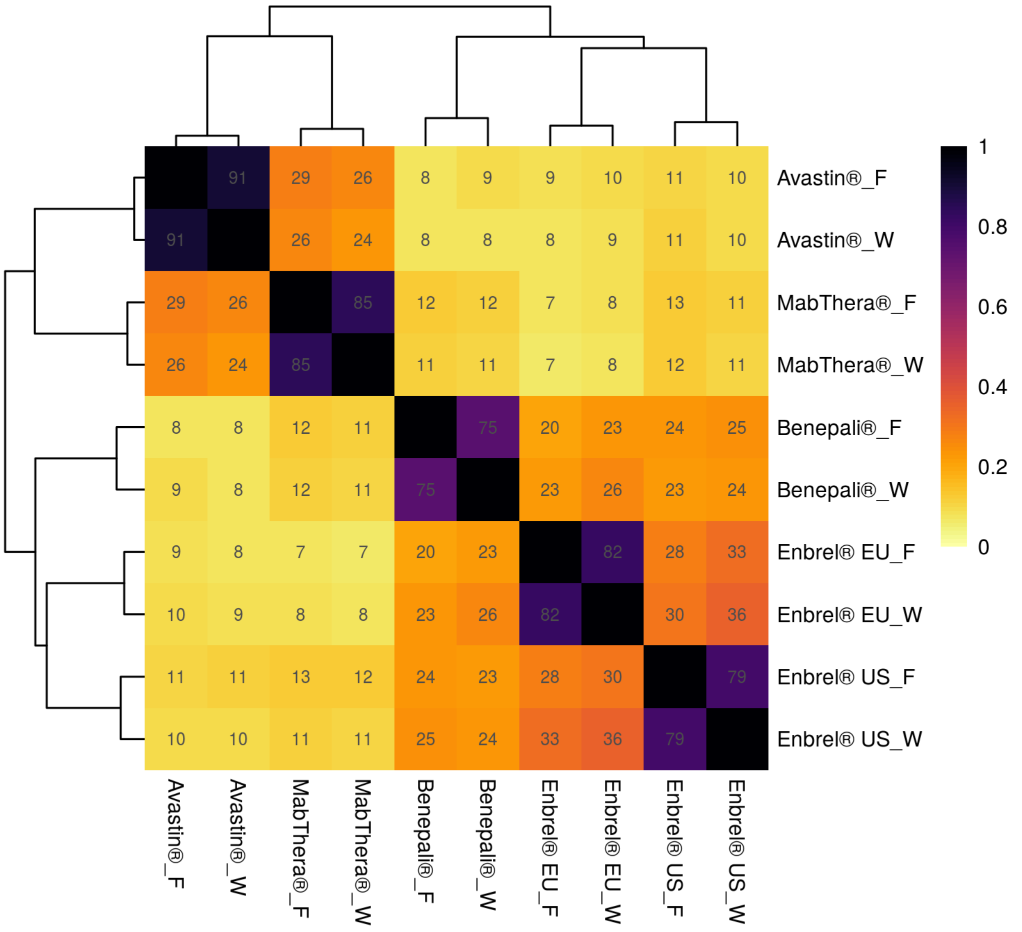


```r
ggsave_default("figure_5b", width = 13.6, height = 12.4)
```

… and replicates.

```r
jaccard_data_hcp %>%
  unite(drug, sample, col = "sample") %>%
  jaccard_matrix(member_col = protein) %>%
  jaccard_heatmap(size = "small")
```

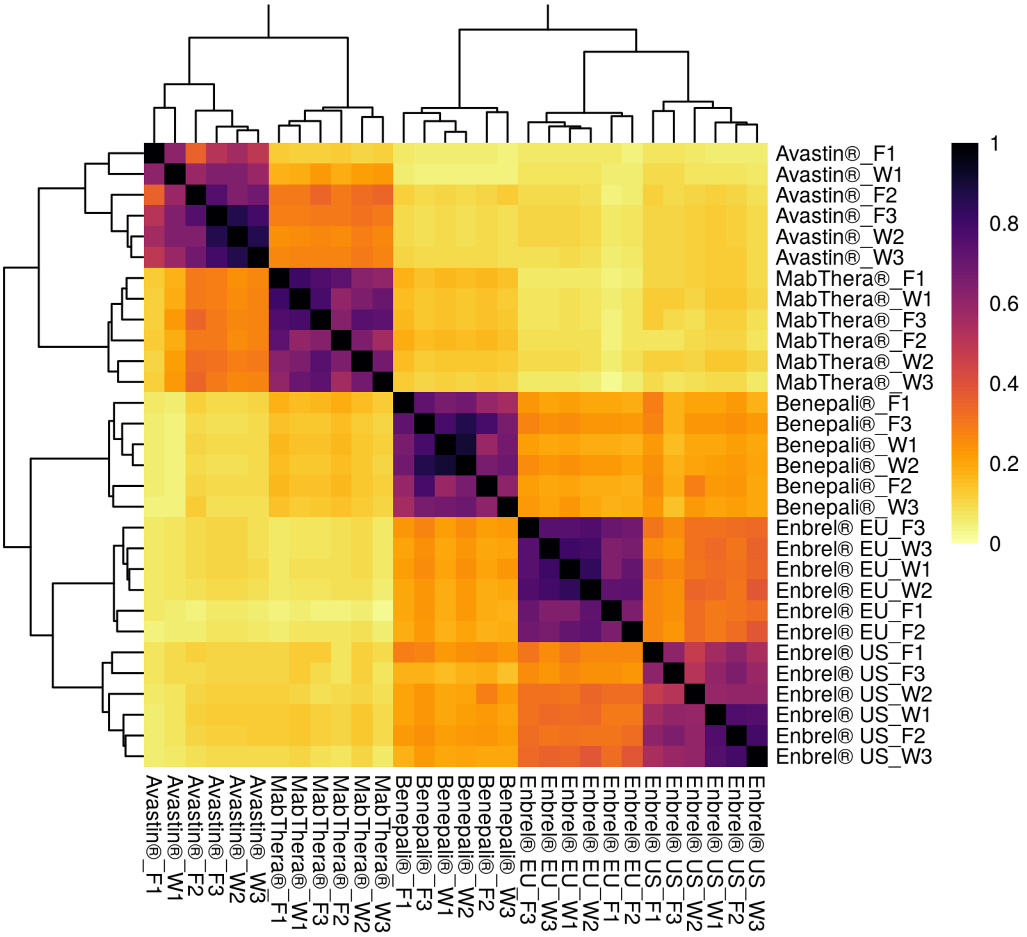


```
ggsave_default("figure_5c", width = 13.6, height = 12.4)
```

Other analyses

(Note: Numbers in the tables below are cited in the *Results and Discussion* section of the manuscript.)

- How many unique peptides were detected in the direct workflow by MS/MS? (subsection *Direct HCP identification …*, second paragraph)

```
peptide_abundances_workflow %>%
  count_peptides() %>%
  filter(
    sample == "D",
    credibility == "MS/MS",
    uniqueness == "unique"
```

```
  ) %>%
  group_by(drug, sample) %>%
  summarise(n = sum(n)) %>%
  arrange(sample, n) %>%
  knitr::kable()
```

drug	sample	n
Enbrel® EU	D	45
Enbrel® US	D	53
Benepali®	D	56
Avastin®	D	80
MabThera®	D	103

- What is the corresponding number of HCPs with at least one unique peptide? (*ibidem*)

```
hcp_abundances_workflow %>%
  count_hcps() %>%
  filter(
    sample == "D",
    credibility %>% str_starts("MS/MS, ")
  ) %>%
  group_by(drug, sample) %>%
  summarise(n = sum(n)) %>%
  arrange(n) %>%
  knitr::kable()
```

drug	sample	n
MabThera®	D	16
Enbrel® EU	D	17
Avastin®	D	18
Enbrel® US	D	18
Benepali®	D	23

- How many unique HCP-derived peptides were detected by MS/MS? (*ibidem*)

```
peptide_abundances_workflow %>%
  count_peptides() %>%
  filter(
    sample == "D",
```

```
    type == "HCPs",
    credibility == "MS/MS",
    uniqueness == "unique"
  ) %>%
  group_by(drug, sample) %>%
  summarise(n = sum(n)) %>%
  arrange(n) %>%
  knitr::kable()
```

drug	sample	n
Enbrel® EU	D	14
Enbrel® US	D	19
Benepali®	D	22
MabThera®	D	25
Avastin®	D	28

- What is the corresponding number of HCPs with at least one unique peptide? (*ibidem*)

```
hcp_abundances_workflow %>%
  count_hcps() %>%
  filter(
    sample == "D",
    type == "HCPs",
    credibility %>% str_starts("MS/MS, ")
  ) %>%
  group_by(drug, sample) %>%
  summarise(n = sum(n)) %>%
  arrange(n) %>%
  knitr::kable()
```

drug	sample	n
MabThera®	D	9
Avastin®	D	11
Enbrel® EU	D	13
Enbrel® US	D	14
Benepali®	D	19

- How many unique HCP-derived peptides were detected in the direct workflow by MS/MS? (subsection *HCP identification upon drug molecule depletion*, second

paragraph)

```
peptide_abundances_workflow %>%
  count_peptides() %>%
  filter(
    sample == "F+W",
    type == "HCPs",
    credibility == "MS/MS",
    uniqueness == "unique"
  ) %>%
  group_by(drug, sample) %>%
  summarise(n = sum(n)) %>%
  arrange(n) %>%
  knitr::kable()
```

drug	sample	n
MabThera®	F+W	52
Benepali®	F+W	74
Enbrel® US	F+W	120
Avastin®	F+W	125
Enbrel® EU	F+W	218

- What is the corresponding number of HCPs with at least one unique peptide? (*ibidem*)

```
hcp_abundances_workflow %>%
  count_hcps() %>%
  filter(
    sample == "F+W",
    type == "HCPs",
    credibility %>% str_starts("MS/MS, ")
  ) %>%
  group_by(drug, sample) %>%
  summarise(n = sum(n)) %>%
  arrange(n) %>%
  knitr::kable()
```

drug	sample	n
Benepali®	F+W	22
MabThera®	F+W	23
Avastin®	F+W	29

drug	sample	n
Enbrel® US	F+W	43
Enbrel® EU	F+W	64

- How many shared peptides were considered by the probabilistic algorithm? (subsection *Improved HCP identification ...*, second paragraph)

```
peptide_abundances_workflow %>%
  count_peptides() %>%
  filter(
    sample == "F+W",
    type == "HCPs",
    credibility == "MS/MS",
    uniqueness == "shared"
  ) %>%
  group_by(drug, sample) %>%
  arrange(n) %>%
  knitr::kable()
```

drug	sample	type	credibility	uniqueness	n
Enbrel® US	F+W	HCPs	MS/MS	shared	10
Benepali®	F+W	HCPs	MS/MS	shared	15
MabThera®	F+W	HCPs	MS/MS	shared	16
Avastin®	F+W	HCPs	MS/MS	shared	19
Enbrel® EU	F+W	HCPs	MS/MS	shared	29

- How many HCPs were additionally identified by the probabilistic algorithm? (*ibidem*)

```
hcp_abundances_workflow %>%
  count_hcps() %>%
  filter(
    sample == "F+W",
    type == "HCPs",
    credibility == "MS/MS"
  ) %>%
  arrange(sample, credibility, n) %>%
  knitr::kable()
```

drug	sample	type	credibility	n
Avastin®	F+W	HCPs	MS/MS	1
MabThera®	F+W	HCPs	MS/MS	2
Benepali®	F+W	HCPs	MS/MS	2
Enbrel® EU	F+W	HCPs	MS/MS	3
Enbrel® US	F+W	HCPs	MS/MS	3

- How many *in silico* peptides were detected? (third paragraph)

```
peptide_abundances_workflow %>%
  count_peptides() %>%
  filter(
    sample == "F+W",
    type == "HCPs",
    credibility == "In silico"
  ) %>%
  group_by(drug, sample) %>%
  summarise(n = sum(n)) %>%
  arrange(sample, n) %>%
  knitr::kable()
```

drug	sample	n
Enbrel® EU	F+W	0
Benepali®	F+W	5
Enbrel® US	F+W	7
MabThera®	F+W	10
Avastin®	F+W	17

- How many HCPs were additionally identified by these *in silico* peptides? (*ibidem*)

```
hcp_abundances_workflow %>%
  count_hcps() %>%
  filter(
    sample == "F+W",
    type == "HCPs",
    credibility == "In silico"
  ) %>%
  arrange(sample, n) %>%
  knitr::kable()
```

drug	sample	type	credibility	n
Enbrel® EU	F+W	HCPs	In silico	0
MabThera®	F+W	HCPs	In silico	1
Enbrel® US	F+W	HCPs	In silico	2
Avastin®	F+W	HCPs	In silico	3
Benepali®	F+W	HCPs	In silico	4

- What is the total number of HCPs identified in this study? (subsection *Comparative analysis of HCP profiles*, first paragraph)

```
hcp_abundances %>%
  filter(
    type == "HCPs",
    !str_starts(sample, "D")
  ) %>%
  pull(protein) %>%
  n_distinct()
#> [1] 127
```

- What is the total number of corresponding peptides? (*ibidem*)

```
peptide_abundances %>%
  filter(
    type == "HCPs",
    !str_starts(sample, "D")
  ) %>%
  pull(sequence) %>%
  n_distinct()
#> [1] 537
```

- Which HCPs have been detected in all drug products?

```
protein_data %>%
  inner_join(
    hcp_abundances_workflow %>%
      filter(sample == "F+W", type == "HCPs") %>%
      group_by(protein) %>%
      summarise(n_drugs = n_distinct(drug)) %>%
      filter(n_drugs == 5),
    by = c(entry = "protein")
  ) %>%
  select(entry, name) %>%
  knitr::kable()
```

entry	name
G3HAC6	Titin
G3HG75	Nestin
G3HL18	Anionic trypsin-2

- By how much do median Jaccard indices increase if peptide and HCP identifications with less credibility are included for assessing repeatability? (third paragraph)

```
hcp_repeatability %>%
  filter(fraction != "D") %>%
  group_by(min_credibility) %>%
  summarise(
    median = median(jaccard_index),
    IQR = IQR(jaccard_index)
  ) %>%
  head(4) %>%
  knitr::kable(digits = 2)
```

min_credibility	median	IQR
In silico	0.68	0.14
MS/MS	0.51	0.25
MS/MS, 1 unique peptide	0.49	0.23
MS/MS, 2 unique peptides	0.39	0.19

Code for supporting figures

Figure S1: Retention time stability

Draw a histogram that summarizes relative standard deviations of peptide retentions times.

```
peptide_data %>%
  group_by(sequence_unformatted, mod_names) %>%
  summarise(sd = sd(obs_time_var_min) / mean(obs_time_var_min) * 100) %>%
  ungroup() %>%
  filter(!is.na(sd)) %>%
  mutate(sd = pmin(sd, 20)) %>%
  ggplot(aes(sd)) +
  geom_histogram(binwidth = 1) +
  scale_x_continuous(
    name = "Relative standard deviation\nof retention time (%)",
    labels = function(x) {
      x[length(x)] <- str_glue("{x[length(x)]} or higher")
      x
    }
  ) +
  ylab("Number of observations")
```

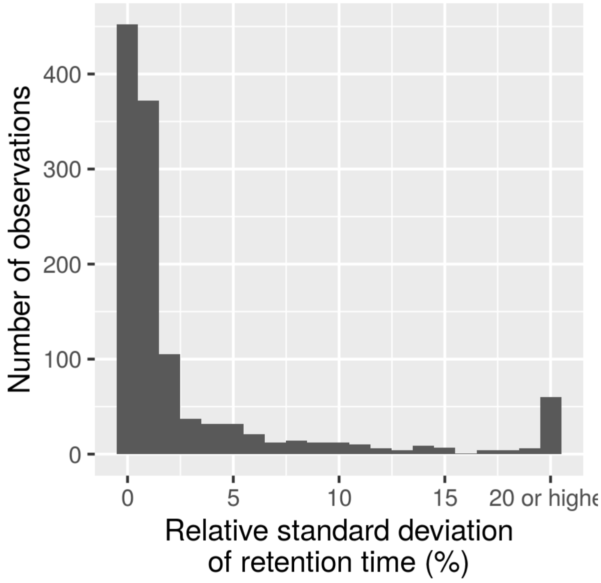


```
ggsave_default("figure_S1", width = 8, height = 8)
```

Figure S2a: PPM distribution

Plot the distribution of deviations between theoretical and experimental peptide masses, as measured in PPM.

```
q2.5_label <- str_glue(
  "2.5th\n({format(insilico_limits$q2.5_ppm, digits = 3)} ppm)"
)
q97.5_label <- str_glue(
  "97.5th percentile\n({format(insilico_limits$q97.5_ppm, digits = 3)} ppm)"
)

peptide_data_raw %>%
  mutate(
    in_silico =
      case_when(
        in_silico ~ "In silico",
        TRUE      ~ "MS/MS"
      ) %>%
      as_factor() %>%
      fct_rev()
  ) %>%
  ggplot() +
  geom_histogram(aes(ppm), binwidth = 2, fill = "#d6604d") +
  geom_vline(
    xintercept = c(insilico_limits$q2.5_ppm, insilico_limits$q97.5_ppm),
    size = .25,
    linetype = "dashed"
  ) +
  geom_text(
    aes(x, y, label = label, hjust = hjust),
    data = tribble(
      ~x,   ~y, ~label,                         ~in_silico,  ~hjust,
      -8, 6850, q2.5_label,                     "MS/MS",          1,
       5, 6850, q97.5_label,                    "MS/MS",          0,
      -8, 2200, "2.5th",                        "In silico",      1,
       5, 2200, "97.5th percentile\nfor MS/MS", "In silico",      0
    ) %>%
      mutate(in_silico = factor(in_silico) %>% fct_rev()),
    vjust = 1
  ) +
  xlab("Mass deviation (ppm)") +
  ylab("Number of identifications") +
  facet_wrap(vars(in_silico), scales = "free_y")
```

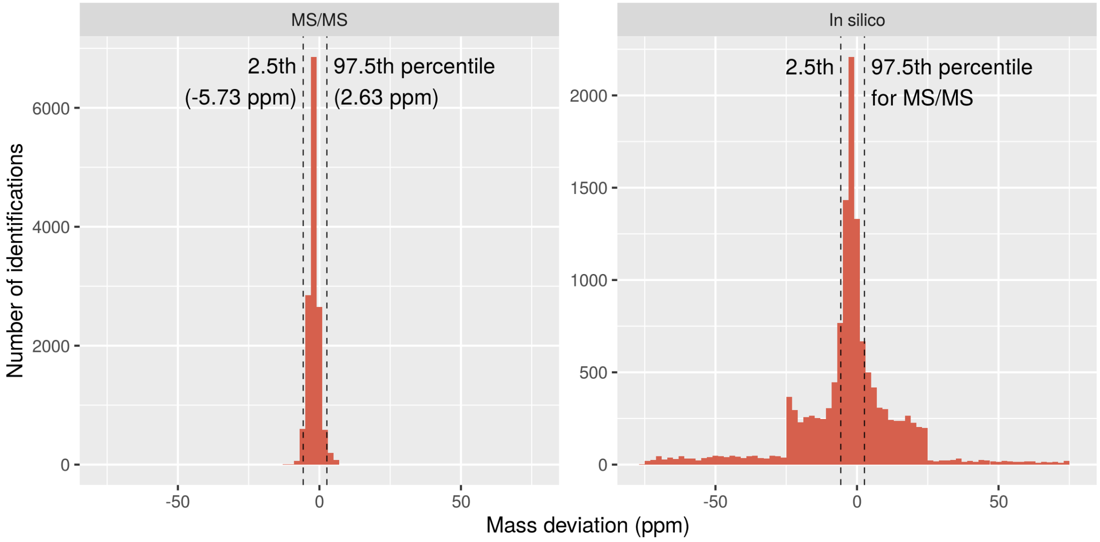


```
ggsave_default("figure_S2a", width = 20, height = 10)
```

Figure S2b: MS1 correlation distribution

Plot the distribution of MS1 correlation coefficients for peptides detected on the MS/MS or full-scan MS level, respectively.

```
peptide_data_raw %>%
  mutate(
    in_silico =
      case_when(
        in_silico ~ "In silico",
        TRUE      ~ "MS/MS"
      ) %>%
      as_factor() %>%
      fct_rev()
  ) %>%
  ggplot() +
  geom_histogram(
    aes(ms1_correlation),
    binwidth = .02,
    fill = "#4393c3"
  ) +
  geom_vline(
    xintercept = insilico_limits$q5_ms1cor,
    size = .25,
    linetype = "dashed"
```

```
) +
geom_text(
  aes(x, y, label = label),
  data = tibble(
    x = .77,
    y = c(4200, 1900),
    label = c(
      str_glue("5th percentile\n",
               "({format(insilico_limits$q5_ms1cor, digits = 3)})"),
      "5th percentile\nfor MS/MS"
    ),
    in_silico = factor(c("MS/MS", "In silico")) %>% fct_rev()),
  hjust = 1,
  vjust = 1
) +
facet_wrap(vars(in_silico), scales = "free_y") +
scale_x_continuous(
  name = "Pearson correlation coefficient",
  labels = function(x)
    case_when(
      near(x, 0) ~ "0 or lower",
      TRUE       ~ as.character(x)
    )
) +
ylab("Number of identifications")
```

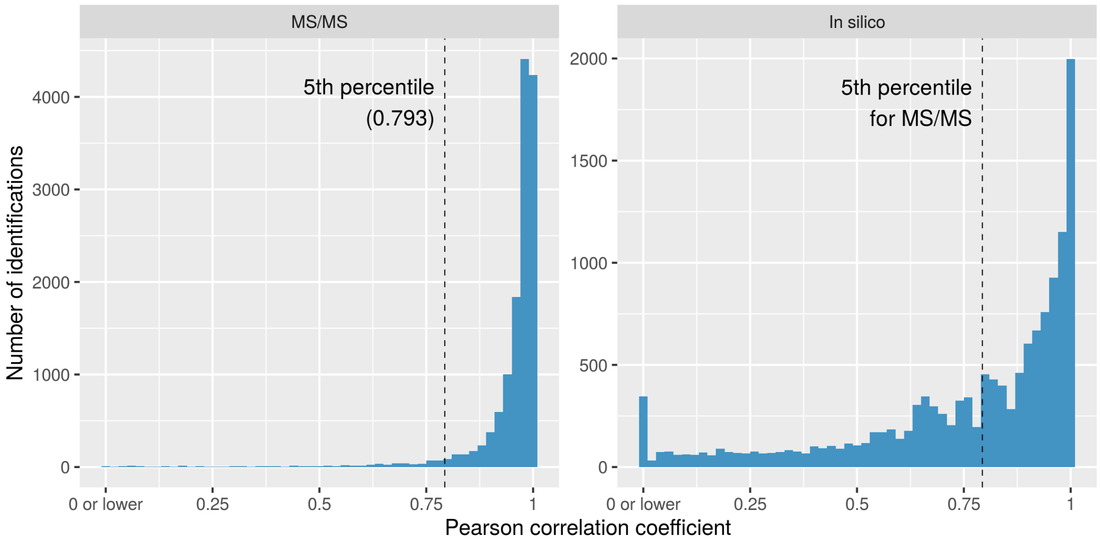

```
ggsave_default("figure_S2b", width = 20, height = 10)
```

Figure S3a: Peptide credibility

Draw a heatmap displaying credibilities for all peptides.

```
draw_peptide_credibility <- function(df) {
  peptide_order <-
    df %>%
    group_by(sequence) %>%
    summarise(
      y_order = sum(credibility == "MS/MS"),
      type = first(type)
    ) %>%
    arrange(desc(type), desc(y_order))

  type_counts <-
    peptide_abundances %>%
    group_by(type) %>%
    summarise(n = n_distinct(sequence)) %>%
    deframe()

  df %>%
    mutate(
      sequence =
        sequence %>%
        as_factor() %>%
        fct_relevel(peptide_order %>% pull(sequence)),
      type = type %>% fct_relabel(~str_glue("{.} ({type_counts[.]})"))
    ) %>%
    ggplot(aes(sample, sequence)) +
    geom_tile(aes(fill = credibility)) +
    scale_x_discrete(name = "", expand = expansion()) +
    scale_y_discrete(
      name = str_glue(
        "{n_distinct(df$sequence)} peptides"
      ),
      expand = expansion(),
      breaks = NULL,
      labels = NULL
    ) +
    scale_fill_manual(
      name = "credibility",
```

```r
      values = c("#F0E442", "#56B4E9")
    ) +
    facet_grid(
      vars(type),
      vars(drug),
      scales = "free_y",
      space = "free_y",
      switch = "both"
    ) +
    theme_bw() +
    theme(
      panel.border = element_blank(),
      panel.background = element_rect(fill = "#f2f2f2"),
      panel.grid = element_blank(),
      panel.spacing.x = unit(2, "mm"),
      strip.background = element_blank(),
      strip.placement = "outside",
      strip.switch.pad.wrap = unit(0, "mm"),
      legend.position = "right"
    )
}

peptide_abundances_fractions %>%
  draw_peptide_credibility()
```

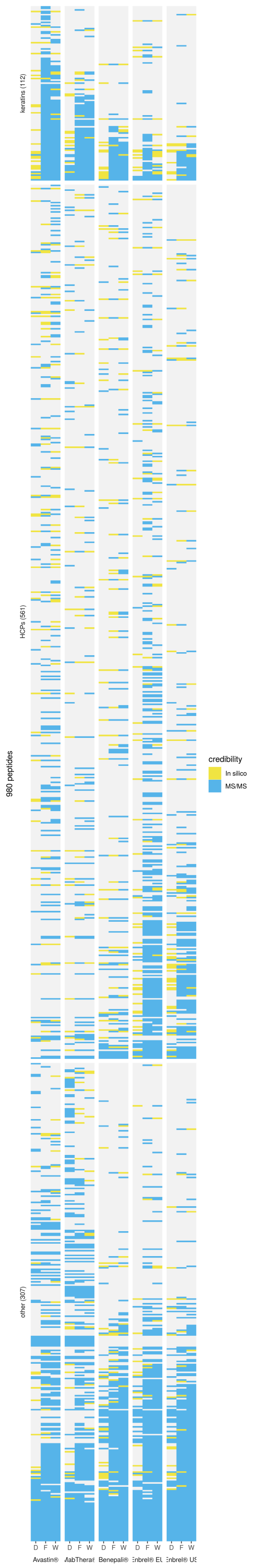
980 peptides
keratins (112)
HCPs (561)
other (307)
credibility
In silico
MS/MS
D F W
D F W
D F W
D F W
D F W
Avastin®
MabThera
Benepali®
nbrel® EU
nbrel® US

```
ggsave_default("figure_S3a", width = 12, height = 75)
```

Figure S3b: HCP credibility

Draw a heatmap displaying credibilities for all proteins.

```
draw_hcp_credibility <- function(df) {
  true_hcps <-
    df %>%
    pull(protein) %>%
    unique() %>%
    setdiff(keratins) %>%
    setdiff(non_hcps) %>%
    sort()

  y_order <-
    c(non_hcps, true_hcps, keratins)

  df %>%
    mutate(
      protein =
        protein %>%
        as_factor() %>%
        fct_relevel(y_order) %>%
        as.numeric()
    ) %>%
    ggplot(aes(sample, protein)) +
    geom_tile(aes(fill = credibility)) +
    scale_x_discrete(name = "", expand = expansion()) +
    scale_y_continuous(
      name = "",
      breaks = seq_along(y_order),
      labels = y_order,
      expand = expansion(),
      sec.axis = dup_axis(
        name = "",
        labels =
          tibble(entry = y_order) %>%
          left_join(protein_data, by = "entry") %>%
          pull(name) %>%
          replace_na("") %>%
          str_sub(end = 80)
      )
```

```r
    ) +
    scale_fill_manual(
      name = "credibility",
      values = color_scale_credibility_hcp,
      drop = FALSE,
    ) +
    facet_grid(
      vars(type),
      vars(drug),
      scales = "free_y",
      space = "free_y",
      switch = "both"
    ) +
    theme_bw() +
    theme(
      axis.ticks.y.right = element_blank(),
      panel.border = element_blank(),
      panel.background = element_rect(fill = "#f2f2f2"),
      panel.grid = element_blank(),
      panel.spacing.x = unit(2, "mm"),
      strip.background = element_blank(),
      strip.placement = "outside",
      strip.switch.pad.wrap = unit(0, "mm"),
      legend.position = c(1.6, 0.015)
    )
}

hcp_abundances_fractions %>%
  draw_hcp_credibility()
```

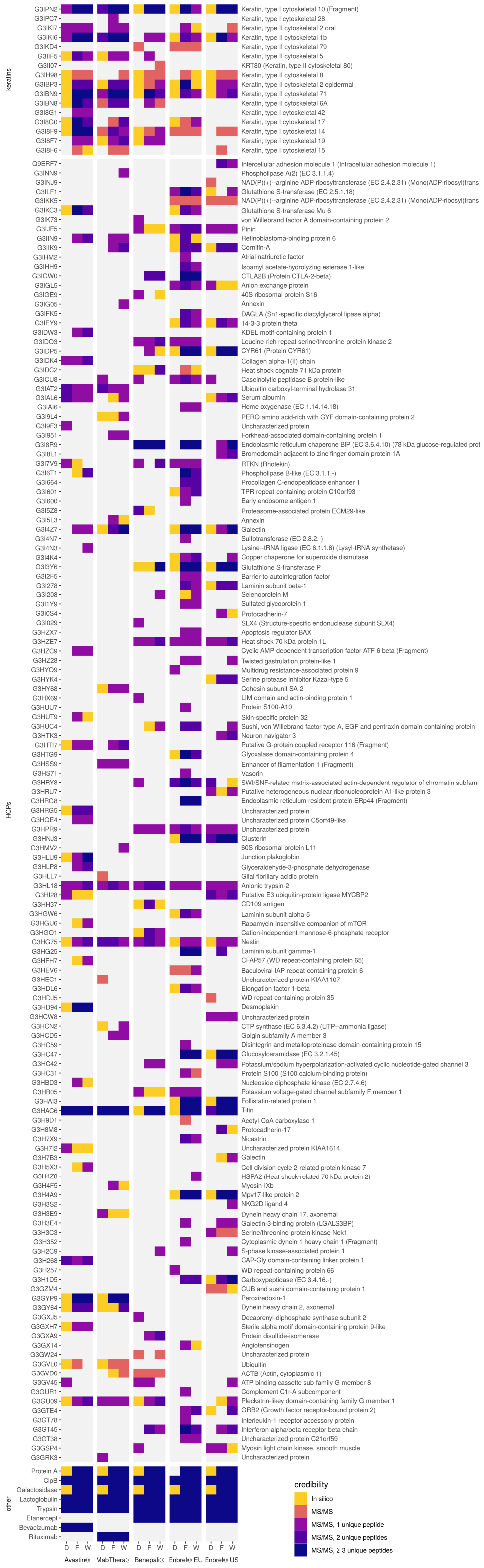
keratins
HCPs
other
Keratin, type I cytoskeletal 10 (Fragment)
Keratin, type I cytoskeletal 28
Keratin, type II cytoskeletal 2 oral
Keratin, type II cytoskeletal 1b
Keratin, type II cytoskeletal 79
Keratin, type II cytoskeletal 5
KRT80 (Keratin, type II cytoskeletal 80)
Keratin, type II cytoskeletal 8
Keratin, type II cytoskeletal 2 epidermal
Keratin, type II cytoskeletal 71
Keratin, type II cytoskeletal 6A
Keratin, type I cytoskeletal 42
Keratin, type I cytoskeletal 17
Keratin, type I cytoskeletal 14
Keratin, type I cytoskeletal 19
Keratin, type I cytoskeletal 15
Intercellular adhesion molecule 1 (Intracellular adhesion molecule 1)
Phospholipase A(2) (EC 3.1.1.4)
NAD(P)(+)--arginine ADP-ribosyltransferase (EC 2.4.2.31) (Mono(ADP-ribosyl)trans
Glutathione S-transferase (EC 2.5.1.18)
NAD(P)(+)--arginine ADP-ribosyltransferase (EC 2.4.2.31) (Mono(ADP-ribosyl)trans
Glutathione S-transferase Mu 6
von Willebrand factor A domain-containing protein 2
Pinin
Retinoblastoma-binding protein 6
Cornifin-A
Atrial natriuretic factor
Isoamyl acetate-hydrolyzing esterase 1-like
CTLA2B (Protein CTLA-2-beta)
Anion exchange protein
40S ribosomal protein S16
Annexin
DAGLA (Sn1-specific diacylglycerol lipase alpha)
14-3-3 protein theta
KDEL motif-containing protein 1
Leucine-rich repeat serine/threonine-protein kinase 2
CYR61 (Protein CYR61)
Collagen alpha-1(II) chain
Heat shock cognate 71 kDa protein
Caseinolytic peptidase B protein-like
Ubiquitin carboxyl-terminal hydrolase 31
Serum albumin
Heme oxygenase (EC 1.14.14.18)
PERQ amino acid-rich with GYF domain-containing protein 2
Uncharacterized protein
Forkhead-associated domain-containing protein 1
Endoplasmic reticulum chaperone BiP (EC 3.6.4.10) (78 kDa glucose-regulated prot
Bromodomain adjacent to zinc finger domain protein 1A
RTKN (Rhotekin)
Phospholipase B-like (EC 3.1.1.-)
Procollagen C-endopeptidase enhancer 1
TPR repeat-containing protein C10orf93
Early endosome antigen 1
Proteasome-associated protein ECM29-like
Annexin
Galectin
Sulfotransferase (EC 2.8.2.-)
Lysine--tRNA ligase (EC 6.1.1.6) (Lysyl-tRNA synthetase)
Copper chaperone for superoxide dismutase
Glutathione S-transferase P
Barrier-to-autointegration factor
Laminin subunit beta-1
Selenoprotein M
Sulfated glycoprotein 1
Protocadherin-7
SLX4 (Structure-specific endonuclease subunit SLX4)
Apoptosis regulator BAX
Heat shock 70 kDa protein 1L
Cyclic AMP-dependent transcription factor ATF-6 beta (Fragment)
Twisted gastrulation protein-like 1
Multidrug resistance-associated protein 9
Serine protease inhibitor Kazal-type 5
Cohesin subunit SA-2
LIM domain and actin-binding protein 1
Protein S100-A10
Skin-specific protein 32
Sushi, von Willebrand factor type A, EGF and pentraxin domain-containing protein
Neuron navigator 3
Putative G-protein coupled receptor 116 (Fragment)
Glyoxalase domain-containing protein 4
Enhancer of filamentation 1 (Fragment)
Vasorin
SWI/SNF-related matrix-associated actin-dependent regulator of chromatin subfami
Putative heterogeneous nuclear ribonucleoprotein A1-like protein 3
Endoplasmic reticulum resident protein ERp44 (Fragment)
Uncharacterized protein
Uncharacterized protein C5orf49-like
Uncharacterized protein
Clusterin
60S ribosomal protein L11
Junction plakoglobin
Glyceraldehyde-3-phosphate dehydrogenase
Glial fibrillary acidic protein
Anionic trypsin-2
Putative E3 ubiquitin-protein ligase MYCBP2
CD109 antigen
Laminin subunit alpha-5
Rapamycin-insensitive companion of mTOR
Cation-independent mannose-6-phosphate receptor
Nestin
Laminin subunit gamma-1
CFAP57 (WD repeat-containing protein 65)
Baculoviral IAP repeat-containing protein 6
Uncharacterized protein KIAA1107
Elongation factor 1-beta
WD repeat-containing protein 35
Desmoplakin
Uncharacterized protein
CTP synthase (EC 6.3.4.2) (UTP--ammonia ligase)
Golgin subfamily A member 3
Disintegrin and metalloproteinase domain-containing protein 15
Glucosylceramidase (EC 3.2.1.45)
Potassium/sodium hyperpolarization-activated cyclic nucleotide-gated channel 3
Protein S100 (S100 calcium-binding protein)
Nucleoside diphosphate kinase (EC 2.7.4.6)
Potassium voltage-gated channel subfamily F member 1
Follistatin-related protein 1
Titin
Acetyl-CoA carboxylase 1
Protocadherin-17
Nicastrin
Uncharacterized protein KIAA1614
Galectin
Cell division cycle 2-related protein kinase 7
HSPA2 (Heat shock-related 70 kDa protein 2)
Myosin-IXb
Mpv17-like protein 2
NKG2D ligand 4
Dynein heavy chain 17, axonemal
Galectin-3-binding protein (LGALS3BP)
Serine/threonine-protein kinase Nek1
Cytoplasmic dynein 1 heavy chain 1 (Fragment)
S-phase kinase-associated protein 1
CAP-Gly domain-containing linker protein 1
WD repeat-containing protein 66
Carboxypeptidase (EC 3.4.16.-)
CUB and sushi domain-containing protein 1
Peroxiredoxin-1
Dynein heavy chain 2, axonemal
Decaprenyl-diphosphate synthase subunit 2
Sterile alpha motif domain-containing protein 9-like
Protein disulfide-isomerase
Angiotensinogen
Uncharacterized protein
Ubiquitin
ACTB (Actin, cytoplasmic 1)
ATP-binding cassette sub-family G member 8
Complement C1r-A subcomponent
Pleckstrin-likey domain-containing family G member 1
GRB2 (Growth factor receptor-bound protein 2)
Interleukin-1 receptor accessory protein
Interferon alpha/beta receptor beta chain
Uncharacterized protein C21orf59
Myosin light chain kinase, smooth muscle
Uncharacterized protein
Protein A
ClpB
Galactosidase
Lactoglobulin
Trypsin
Etanercept
Bevacizumab
Rituximab
D F W
Avastin®
MabThera®
Benepali®
Enbrel® EU
Enbrel® US
credibility
in silico
MS/MS
MS/MS, 1 unique peptide
MS/MS, 2 unique peptides
MS/MS, ≥ 3 unique peptides

```r
ggsave_default("figure_S3b", width = 24, height = 75)
```

Figure S4: Distribution over drugs

`get_distribution()` determines the number of drugs in which a given HCP or peptide has been detected.

```r
get_distribution <- function(df, member_col) {
  df %>%
    filter(sample == "F+W", type == "HCPs") %>%
    group_by({{member_col}}) %>%
    summarise(n_drugs = n_distinct(drug)) %>%
    count(n_drugs)
}

distribution_over_drugs <-
  bind_rows(
    HCPs =
      hcp_abundances_workflow %>%
      get_distribution(protein),
    Peptides =
      peptide_abundances_workflow %>%
      get_distribution(sequence),
    .id = "type"
  )
```

The following HCPs have been detected in at least four drugs:

```r
protein_data %>%
  inner_join(
    hcp_abundances_workflow %>%
      filter(sample == "F+W", type == "HCPs") %>%
      group_by(protein) %>%
      summarise(n_drugs = n_distinct(drug)) %>%
      filter(n_drugs >= 4),
    by = c(entry = "protein")
  ) %>%
  select(entry, n_drugs) %>%
  arrange(n_drugs) %>%
  knitr::kable()
```

entry	n_drugs
G3GU09	4
G3I4Z7	4
G3HAC6	5
G3HG75	5
G3HL18	5

The following peptides have been detected in at least four drugs:

```
peptide_abundances_workflow %>%
  filter(sample == "F+W", type == "HCPs") %>%
  group_by(sequence) %>%
  summarise(n_drugs = n_distinct(drug)) %>%
  filter(n_drugs >= 4) %>%
  arrange(n_drugs) %>%
  knitr::kable()
```

sequence	n_drugs
SFVLNLGK	4
AALITETLQLSR	5
TLDNDIMLIKLASPVTLNSR	5
TLLEAENSR	5

Draw a bar chart summarizing this data.

```
distribution_over_drugs %>%
  ggplot(aes(n_drugs, n)) +
  geom_col(aes(fill = factor(n_drugs)), show.legend = FALSE) +
  scale_y_continuous(
    name = "Number of identifications",
    expand = expansion(c(0, .05))
  ) +
  scale_fill_brewer(palette = "RdPu") +
  facet_wrap(vars(type), scales = "free_y") +
  xlab("Number of drug products with identifications") +
  theme_bw() +
  theme(
    axis.ticks.x = element_blank(),
    panel.border = element_blank(),
    panel.grid.major.x = element_blank(),
    panel.grid.minor.x = element_blank(),
```

```
    panel.grid.minor.y = element_blank(),
    strip.background = element_blank(),
    strip.placement = "outside",
    strip.text = element_text(face = "bold")
  )
```

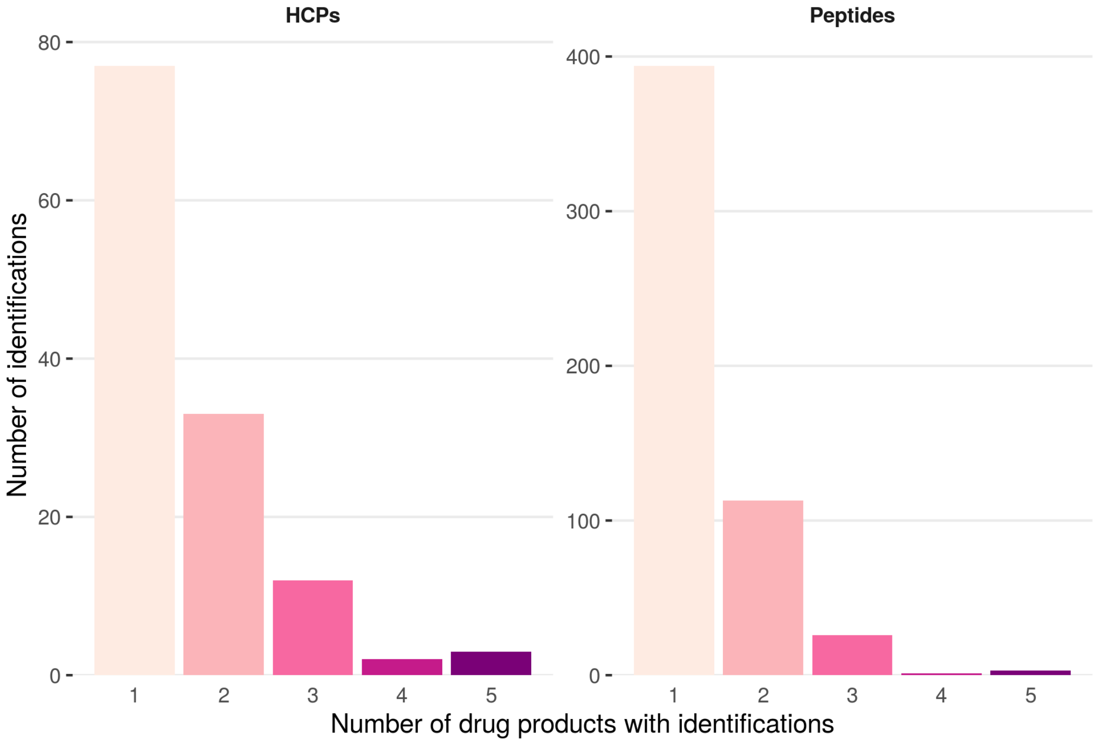


```
ggsave_default("figure_S4", width = 12, height = 10)
```

Figure S5: Overlap flow-through/wash

The following auxiliary functions prepare data for plotting the number of HCPs and peptides shared between the flow-through and wash fractions.

```
get_overlap_data <- function(df, member_col) {
  df %>%
    filter(
      type == "HCPs",
      !str_starts(sample, "D"),
    ) %>%
    mutate(sample = str_c(drug, str_sub(sample, 1, 1))) %>%
    jaccard_matrix(member_col = {{member_col}}, as_matrix = FALSE) %>%
```

```r
    rename(members_f = members_x, members_w = members_y) %>%
    filter(
      str_sub(x, end = -2) == str_sub(y, end = -2),
      str_sub(x, -1, -1) == "F",
      str_sub(y, -1, -1) == "W"
    ) %>%
    mutate(
      drug = str_sub(x, end = -2),
      size_f_exclusive = pmap_int(
        .,
        function(members_f, members_w, ...) {
          members_f %>% setdiff(members_w) %>% length()
        }
      ),
      size_w_exclusive = pmap_int(
        .,
        function(members_f, members_w, ...) {
          members_w %>% setdiff(members_f) %>% length
        }
      ),
    ) %>%
    select(drug, starts_with("size"))
}

get_overlap_data_bar <- function(df) {
  df %>%
    mutate(size_intersection_half = size_intersection / 2) %>%
    pivot_longer(
      -drug,
      names_to = "measure",
      names_prefix = "size_",
      values_to = "n"
    )
}

get_overlap_data_label <- function(df) {
  df %>%
    mutate(
      labelpos_intersection = 0,
      labelpos_w_exclusive = -size_intersection / 2 + 2,
      labelpos_f_exclusive = size_intersection / 2 - 2,
      hjust_intersection = .5,
      hjust_w_exclusive = 0,
```

```
      hjust_f_exclusive = 1,
      color_intersection = "grey40",
      color_w_exclusive = "#d95f02",
      color_f_exclusive = "#1b9e77",
    ) %>%
    select(-size_union) %>%
    pivot_longer(
      -drug,
      names_to = c(".value", "measure"),
      names_pattern = "([^_]+)_(.+)",
      values_to = "value"
    )
}
```

Plot these numbers of overlapping detections.

```
overlap_data_hcp <-
  hcp_abundances %>%
  get_overlap_data(member_col = protein)

overlap_data_peptide <-
  peptide_abundances %>%
  get_overlap_data(member_col = sequence)

overlap_data_bar <-
  bind_rows(
    HCPs =
      overlap_data_hcp %>%
      get_overlap_data_bar(),
    Peptides =
      overlap_data_peptide %>%
      get_overlap_data_bar(),
    .id = "type"
  ) %>%
  recode_dp_names()

overlap_data_label <-
  bind_rows(
    HCPs =
      overlap_data_hcp %>%
      get_overlap_data_label(),
    Peptides =
      overlap_data_peptide %>%
      get_overlap_data_label(),
```

```r
    .id = "type"
  ) %>%
  recode_dp_names()

ggplot(mapping = aes(drug)) +
  geom_col(
    data =
      overlap_data_bar %>%
      filter(measure %in% c("intersection_half", "f_exclusive")),
    aes(y = n, fill = measure),
    width = .45
  ) +
  geom_col(
    data =
      overlap_data_bar %>%
      filter(measure %in% c("intersection_half", "w_exclusive")),
    aes(y = -n, fill = factor(measure) %>% fct_rev()),
    width = .45
  ) +
  geom_text(
    data = overlap_data_label,
    aes(y = labelpos, label = size, hjust = hjust, color = color)
  ) +
  scale_fill_manual(values = c("#1b9e77", "grey92", "#d95f02")) +
  scale_color_identity() +
  coord_flip(clip = "off") +
  facet_wrap(vars(type), scales = "free_x") +
  xlab("") +
  ylab("") +
  theme_bw() +
  theme(
    axis.ticks.x = element_blank(),
    axis.text.x = element_blank(),
    panel.grid = element_blank(),
    strip.background = element_blank(),
    strip.placement = "outside",
    strip.text = element_text(face = "bold")
  )
```

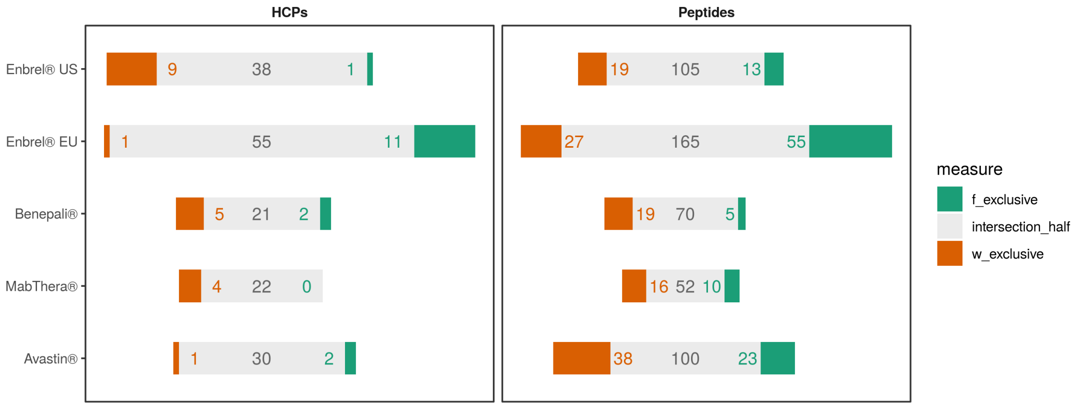


```r
ggsave_default("figure_S5", width = 25, height = 10)
```

Figure S6: Peptide Jaccard plots

Draw heatmaps for comparison of HCP peptide profiles on the level of drugs, …

```r
jaccard_data_peptide <-
  peptide_abundances %>%
  filter(
    type == "HCPs",
    !str_starts(sample, "D")
  )

jaccard_data_peptide %>%
  jaccard_matrix(sample_col = drug, member_col = sequence) %>%
  jaccard_heatmap(size = "large")
```

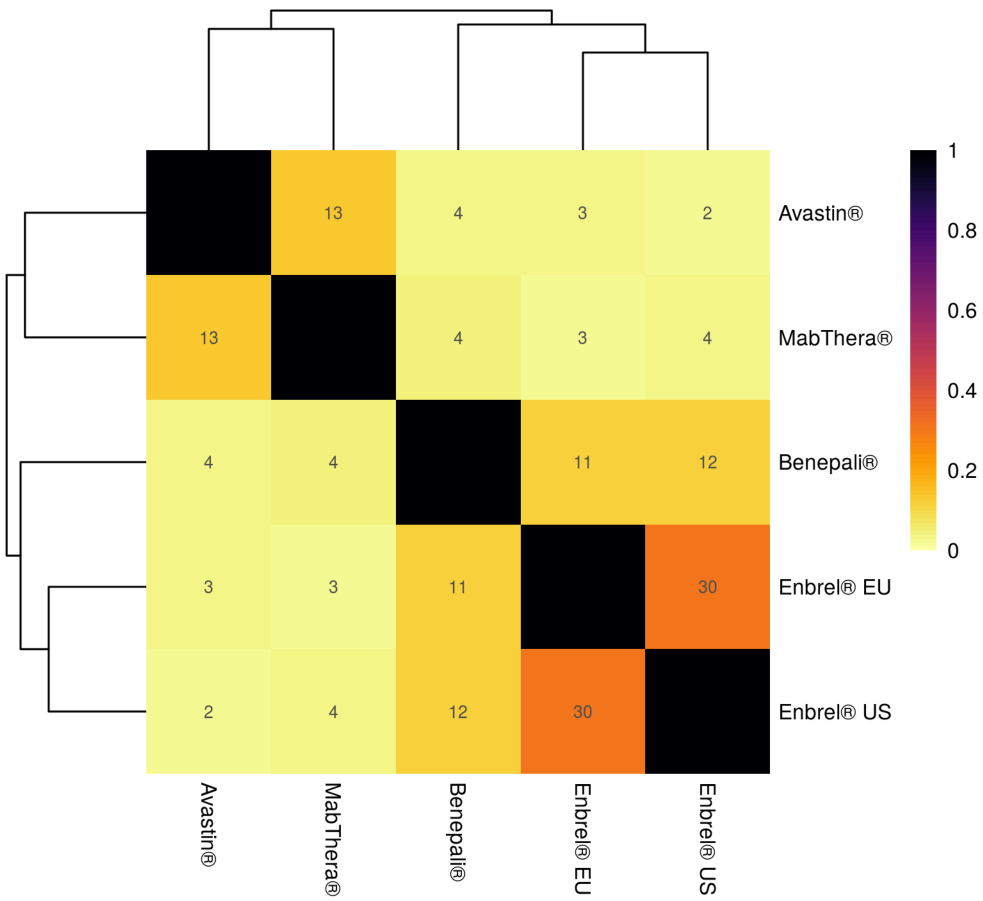


```
ggsave_default("figure_S6a", width = 13.6, height = 12.4)
```

… fractions, …

```
jaccard_data_peptide %>%
  mutate(sample = str_c(drug, str_sub(sample, 1, 1), sep = "_")) %>%
  jaccard_matrix(member_col = sequence) %>%
  jaccard_heatmap(size = "medium")
```

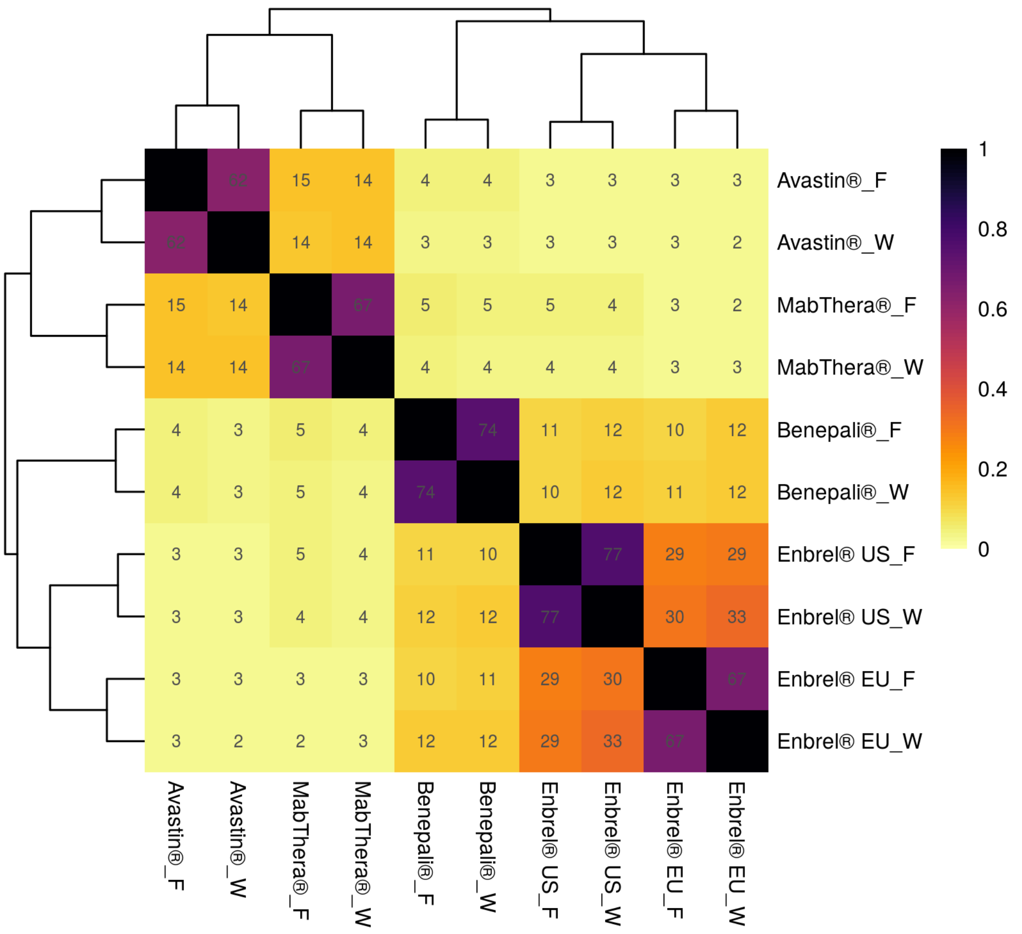


```r
ggsave_default("figure_S6b", width = 13.6, height = 12.4)
```

… and replicates.

```r
jaccard_data_peptide %>%
  unite(drug, sample, col = "sample") %>%
  jaccard_matrix(member_col = sequence) %>%
  jaccard_heatmap(size = "small")
```

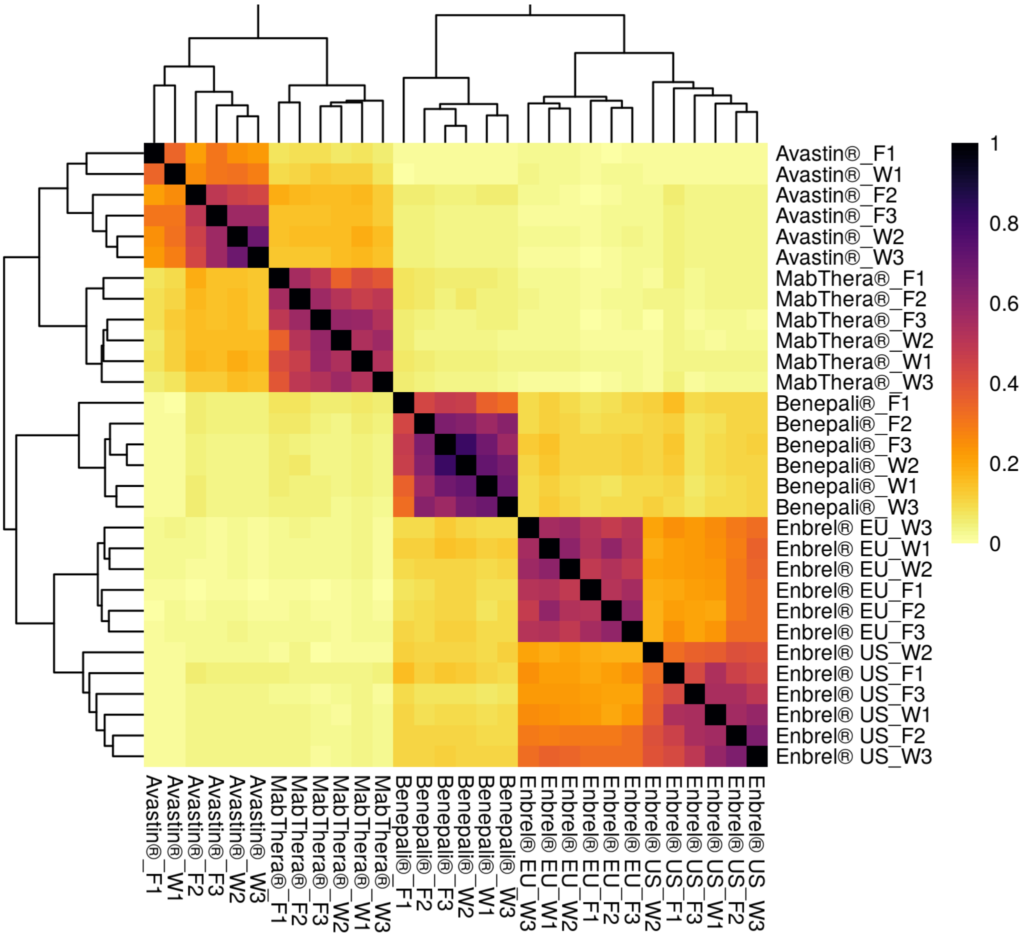


```r
ggsave_default("figure_S6c", width = 13.6, height = 12.4)
```

Session info

This section contains detailed information about the platform and packages that have been used for compiling the analyses.

```
devtools::session_info()
#> - Session info ---------------------------------------------------------------
#>  setting  value
#>  version  R version 3.6.3 (2020-02-29)
#>  os       Linux Mint 18.1
#>  system   x86_64, linux-gnu
#>  ui       X11
#>  language (EN)
#>  collate  en_US.UTF-8
#>  ctype    en_US.UTF-8
#>  tz       Europe/Vienna
#>  date     2020-06-16
#>
#> - Packages -------------------------------------------------------------------
#>  package      * version date       lib source
#>  assertthat     0.2.1   2019-03-21 [1] CRAN (R 3.6.0)
#>  backports      1.1.5   2019-10-02 [1] CRAN (R 3.6.1)
#>  BiocGenerics * 0.30.0  2019-05-02 [1] Bioconductor
#>  Biostrings   * 2.52.0  2019-05-02 [1] Bioconductor
#>  broom          0.5.5   2020-02-29 [1] CRAN (R 3.6.2)
#>  callr          3.4.3   2020-03-28 [1] CRAN (R 3.6.2)
#>  cellranger     1.1.0   2016-07-27 [1] CRAN (R 3.6.0)
#>  cli            2.0.2   2020-02-28 [1] CRAN (R 3.6.2)
#>  codetools      0.2-16  2018-12-24 [4] CRAN (R 3.5.2)
#>  colorspace     1.4-1   2019-03-18 [1] CRAN (R 3.6.0)
#>  crayon         1.3.4   2017-09-16 [1] CRAN (R 3.6.0)
#>  curl           4.3     2019-12-02 [1] CRAN (R 3.6.1)
#>  DBI            1.1.0   2019-12-15 [1] CRAN (R 3.6.1)
#>  dbplyr         1.4.2   2019-06-17 [1] CRAN (R 3.6.0)
#>  desc           1.2.0   2018-05-01 [1] CRAN (R 3.6.0)
#>  devtools       2.2.2   2020-02-17 [1] CRAN (R 3.6.2)
#>  digest         0.6.25  2020-02-23 [1] CRAN (R 3.6.2)
#>  dplyr        * 0.8.5   2020-03-07 [1] CRAN (R 3.6.2)
#>  ellipsis       0.3.0   2019-09-20 [1] CRAN (R 3.6.1)
#>  evaluate       0.14    2019-05-28 [1] CRAN (R 3.6.0)
#>  fansi          0.4.1   2020-01-08 [1] CRAN (R 3.6.2)
#>  farver         2.0.3   2020-01-16 [1] CRAN (R 3.6.1)
#>  forcats      * 0.5.0   2020-03-01 [1] CRAN (R 3.6.2)
#>  fs           * 1.3.2   2020-03-05 [1] CRAN (R 3.6.2)
```

```
#>  gdtools      * 0.2.1   2019-10-14 [1] CRAN (R 3.6.1)
#>  generics       0.0.2   2018-11-29 [1] CRAN (R 3.6.0)
#>  ggplot2      * 3.3.0   2020-03-05 [1] CRAN (R 3.6.2)
#>  glue           1.3.2   2020-03-12 [1] CRAN (R 3.6.2)
#>  gridExtra      2.3     2017-09-09 [1] CRAN (R 3.6.0)
#>  gtable         0.3.0   2019-03-25 [1] CRAN (R 3.6.0)
#>  haven          2.2.0   2019-11-08 [1] CRAN (R 3.6.1)
#>  highr          0.8     2019-03-20 [1] CRAN (R 3.6.0)
#>  hms            0.5.3   2020-01-08 [1] CRAN (R 3.6.1)
#>  htmltools      0.4.0   2019-10-04 [1] CRAN (R 3.6.1)
#>  httr         * 1.4.1   2019-08-05 [1] CRAN (R 3.6.0)
#>  IRanges      * 2.18.1  2019-05-31 [1] Bioconductor
#>  janitor      * 1.2.1   2020-01-22 [1] CRAN (R 3.6.1)
#>  jsonlite       1.6.1   2020-02-02 [1] CRAN (R 3.6.1)
#>  knitr          1.28    2020-02-06 [1] CRAN (R 3.6.1)
#>  labeling       0.3     2014-08-23 [1] CRAN (R 3.6.0)
#>  lattice        0.20-41 2020-04-02 [4] CRAN (R 3.6.3)
#>  lifecycle      0.2.0   2020-03-06 [1] CRAN (R 3.6.2)
#>  lubridate      1.7.4   2018-04-11 [1] CRAN (R 3.6.0)
#>  magrittr       1.5     2014-11-22 [1] CRAN (R 3.6.0)
#>  memoise        1.1.0   2017-04-21 [1] CRAN (R 3.6.0)
#>  modelr         0.1.6   2020-02-22 [1] CRAN (R 3.6.2)
#>  munsell        0.5.0   2018-06-12 [1] CRAN (R 3.6.0)
#>  nlme           3.1-147 2020-04-13 [4] CRAN (R 3.6.3)
#>  pheatmap     * 1.0.12  2019-01-04 [1] CRAN (R 3.6.0)
#>  pillar         1.4.3   2019-12-20 [1] CRAN (R 3.6.1)
#>  pkgbuild       1.0.6   2019-10-09 [1] CRAN (R 3.6.1)
#>  pkgconfig      2.0.3   2019-09-22 [1] CRAN (R 3.6.1)
#>  pkgload        1.0.2   2018-10-29 [1] CRAN (R 3.6.0)
#>  prettyunits    1.1.1   2020-01-24 [1] CRAN (R 3.6.1)
#>  processx       3.4.2   2020-02-09 [1] CRAN (R 3.6.1)
#>  ps             1.3.2   2020-02-13 [1] CRAN (R 3.6.2)
#>  purrr        * 0.3.3   2019-10-18 [1] CRAN (R 3.6.1)
#>  R6             2.4.1   2019-11-12 [1] CRAN (R 3.6.1)
#>  RColorBrewer   1.1-2   2014-12-07 [1] CRAN (R 3.6.0)
#>  Rcpp           1.0.4   2020-03-17 [1] CRAN (R 3.6.2)
#>  readr        * 1.3.1   2018-12-21 [1] CRAN (R 3.6.0)
#>  readxl         1.3.1   2019-03-13 [1] CRAN (R 3.6.0)
#>  remotes        2.1.1   2020-02-15 [1] CRAN (R 3.6.2)
#>  reprex         0.3.0   2019-05-16 [1] CRAN (R 3.6.0)
#>  rlang          0.4.5   2020-03-01 [1] CRAN (R 3.6.2)
#>  rmarkdown      2.1     2020-01-20 [1] CRAN (R 3.6.1)
#>  rprojroot      1.3-2   2018-01-03 [1] CRAN (R 3.6.0)
```

```
#>  rstudioapi      0.11    2020-02-07 [1] CRAN (R 3.6.1)
#>  rvest           0.3.5   2019-11-08 [1] CRAN (R 3.6.1)
#>  S4Vectors     * 0.22.0  2019-05-02 [1] Bioconductor
#>  scales          1.1.0   2019-11-18 [1] CRAN (R 3.6.1)
#>  sessioninfo     1.1.1   2018-11-05 [1] CRAN (R 3.6.0)
#>  snakecase       0.11.0  2019-05-25 [1] CRAN (R 3.6.1)
#>  stringi         1.4.6   2020-02-17 [1] CRAN (R 3.6.2)
#>  stringr       * 1.4.0   2019-02-10 [1] CRAN (R 3.6.0)
#>  svglite         1.2.3   2020-02-07 [1] CRAN (R 3.6.1)
#>  systemfonts     0.1.1   2019-07-01 [1] CRAN (R 3.6.1)
#>  testthat        2.3.2   2020-03-02 [1] CRAN (R 3.6.2)
#>  tibble        * 3.0.0   2020-03-30 [1] CRAN (R 3.6.2)
#>  tidyr         * 1.0.2   2020-01-24 [1] CRAN (R 3.6.1)
#>  tidyselect      1.0.0   2020-01-27 [1] CRAN (R 3.6.1)
#>  tidyverse     * 1.3.0   2019-11-21 [1] CRAN (R 3.6.1)
#>  usethis         1.5.1   2019-07-04 [1] CRAN (R 3.6.0)
#>  vctrs           0.2.4   2020-03-10 [1] CRAN (R 3.6.2)
#>  viridis       * 0.5.1   2018-03-29 [1] CRAN (R 3.6.0)
#>  viridisLite   * 0.3.0   2018-02-01 [1] CRAN (R 3.6.0)
#>  withr           2.1.2   2018-03-15 [1] CRAN (R 3.6.0)
#>  xfun            0.12    2020-01-13 [1] CRAN (R 3.6.1)
#>  xml2            1.2.5   2020-03-11 [1] CRAN (R 3.6.2)
#>  XVector       * 0.24.0  2019-05-02 [1] Bioconductor
#>  yaml            2.2.1   2020-02-01 [1] CRAN (R 3.6.1)
#>  zlibbioc        1.30.0  2019-05-02 [1] Bioconductor
#>
#> [1] /home/wolfgang/Programme/R/3.6
#> [2] /usr/local/lib/R/site-library
#> [3] /usr/lib/R/site-library
#> [4] /usr/lib/R/library
```

References

(1) Shafranovich, Y. *Common Format and Mime Type for Comma-Separated Values (Csv) Files*; RFC 4180; RFC Editor; Internet Requests for Comments; RFC Editor, 2005.

(2) Xie, Y.; Allaire, J.; Grolemund, G. *R Markdown: The Definitive Guide*; Chapman; Hall/CRC: Boca Raton, Florida, 2018.

(3) Xie, Y. *Dynamic Documents with R and Knitr*, 2nd ed.; Chapman; Hall/CRC: Boca Raton, Florida, 2015.

(4) R Core Team. *R: A Language and Environment for Statistical Computing*; R Foundation for Statistical Computing: Vienna, Austria, 2018.

(5) Wickham, H.; Averick, M.; Bryan, J.; Chang, W.; McGowan, L.; François, R.; Grolemund, G.; Hayes, A.; Henry, L.; Hester, J.; Kuhn, M.; Pedersen, T.; Miller, E.; Bache, S.; Müller, K.; Ooms, J.; Robinson, D.; Seidel, D.; Spinu, V.; Takahashi, K.; Vaughan, D.; Wilke, C.; Woo, K.; Yutani, H. Welcome to the Tidyverse. *J. Open Source Softw.* **2019**, *4* (43), 1686.

(6) Amezquita, R. A.; Lun, A. T. L.; Becht, E.; Carey, V. J.; Carpp, L. N.; Geistlinger, L.; Marini, F.; Rue-Albrecht, K.; Risso, D.; Soneson, C.; Waldron, L.; Pagès, H.; Smith, M. L.; Huber, W.; Morgan, M.; Gottardo, R.; Hicks, S. C. Orchestrating Single-Cell Analysis with Bioconductor. *Nat. Methods* **2020**, *17*, 137–145.

(7) Okabe, M.; Ito, K. *Color Universal Design (Cud): How to Make Figures and Presentations That Are Friendly to Colorblind People*; J*FLY, 2008.

(8) Harrower, M. A.; Brewer, C. A. ColorBrewer.org: An Online Tool for Selecting Color Schemes for Maps. *Cartogr. J.* **2003**, *40* (1), 27–37.
